# Epidemiology of gastroesophageal reflux disease in Iran: a systematic review and meta-analysis

**DOI:** 10.1186/s12876-020-01417-6

**Published:** 2020-09-14

**Authors:** Mohammad Karimian, Hassan Nourmohammadi, Majid Salamati, Mohammad Reza Hafezi Ahmadi, Fatemeh Kazemi, Milad Azami

**Affiliations:** 1 Department of General Surgery, Faculty of Medicine, Ilam University of Medical Sciences, Ilam, Iran; 2 Department of Internal Medicine, Shahid Mostafa Khomeini Hospital, Ilam, Iran; 3 Department of Pathology, Faculty of Medicine, Ilam University of Medical Sciences, Ilam, Iran; 4 School of Medicine, Qazvin University of Medical Sciences, Qazvin, Iran; 5 Faculty of Medicine, Ilam University of Medical Sciences, Ilam, Iran

**Keywords:** Epidemiology, Gastroesophageal reflux disease, Iran, Meta-analysis

## Abstract

**Background:**

Gastroesophageal reflux disease (GERD), which leads to acid reflux into the esophagus, is a common gastrointestinal disorder. Several studies have shown the prevalence of GERD in Iranian population, but their evidence is contradictory. Therefore, the present study was conducted to investigate the epidemiology of GERD in Iran.

**Methods:**

The entire steps of this systematic review and meta-analysis were based on the MOOSE protocol, and the results were reported accordance with the PRISMA guideline. This review is registered on PROSPERO (registration number: CRD42020142861). To find potentially relevant published articles, comprehensive search was done on international online databases Scopus, Science Direct, EMBASE, PubMed/Medline, CINAHL, EBSCO, Cochrane Library, Web of Science, Iranian online databases and the Google Scholar search engine in June 2019. Cochran test and I^2^ index were used to assess the heterogeneity of the studies. Data were analyzed using Comprehensive Meta-Analysis software ver. 2. The significance level of the test was considered to be *P* <  0.05.

**Results:**

The daily, weekly, monthly, and overall prevalence of GERD symptoms in Iranian population was 5.64% (95%CI [confidence interval]: 3.77–8.35%; *N* = 66,398), 12.50% (95%CI: 9.63–16.08%; *N* = 110,388), 18.62% (95%CI: 12.90–26.12%; *N* = 70,749) and 43.07% (95%CI: 35.00–51.53%; *N* = 73,189), respectively. The daily, weekly, monthly, and overall prevalence of heartburn in Iranian population was 2.46% (95%CI: 0.93–6.39%; *N* = 18,774), 9.52% (95%CI: 6.16–14.41%; *N* = 54,125), 8.19% (95%CI: 2.42–24.30%; *N* = 19,363) and 23.20% (95%CI: 13.56–36.79%; *N* = 26,543), respectively. The daily, weekly, monthly, and overall prevalence of regurgitation in Iranian population was 4.00% (95%CI: 1.88–8.32%; *N* = 18,774), 9.79% (95%CI: 5.99–15.60%; *N* = 41,140), 13.76% (95%CI: 6.18–44.31%; *N* = 19,363) and 36.53% (95%CI: 19.30–58.08%; *N* = 21,174), respectively. The sensitivity analysis for prevalence of all types GERD, heartburn and regurgitation symptoms by removing a study showed that the overall estimate is still robust.

**Conclusion:**

The present meta-analysis provides comprehensive and useful information on the epidemiology of GERD in Iran for policy-makers and health care providers. This study showed a high prevalence of GERD in Iran. Therefore, effective measures on GERD-related factors such as lifestyle can be among the health policies of Iran.

## Background

Gastroesophageal reflux disease (GERD), which leads to acid reflux into the esophagus, is a common gastrointestinal disorder and results in typical painful symptoms such as heartburn and/or regurgitation [1]. However, it may also appear with atypical symptoms including cough, asthma, chest pain, and fatigue [2].

Permanent acid reflux may cause more severe complications, including erosive esophagitis, esophageal strictures, Barrett’s esophagus, esophageal adenocarcinoma, hiatus hernia, delayed gastric emptying, and visceral hypersensitivity [1, 3–5].

Several risk factors are associated with GERD, including Nonsteroidal Anti-inflammatory Drugs (NSAIDs), type of food, beverages, smoking, family history, high body mass index (BMI), physical activity, salt, or consuming pickles with meals and fast food, which are more associated with the lifestyle of the patient [5–7]. It has also been shown that age, gender, pregnancy, and geographical variation are also related to GERD [7]. In addition, it has been suggested that vertebral fractures and/or spinal malalignment may affect the incidence of GERD [8, 9]. In Iranian studies, consumption of NASIDs and pickle consumption, and smoking is more harmful factors [10, 11].

A systematic review of longitudinal studies suggests that the incidence of GERD has increased in recent decades. If this trend continues, it may rapidly increase the serious complications of GERD, affect the patient’s quality of life, and increase the cost of health care systems [12, 13].

Increasing the GERD awareness to improve Iranian people’s health may be necessary. There is much information in Western cultures that can be generalized to an Iranian person but cannot match completely. Therefore, understanding the epidemiological effects of GERD in Iranian society can help healthcare professionals and policymakers take the next steps in creating the list of priorities for disease management.

Several studies have shown the prevalence of GERD in Iranian population, but their evidence is contradictory [10, 11, 14–39]. Therefore, a structured review of all the documentation and their combination can provide a more complete picture of the dimensions of this disease in Iranian society. One of the main goals of meta-analysis, which is a combination of different studies, is to reduce the difference between parameters due to the increased number of studies involved in the analysis process. Another important goal of meta-analysis is to address inconsistencies in the results and their causes [40–42]. Therefore, the present study was conducted to investigate the epidemiology of GERD in Iran.

## Methods

### Study protocol

The entire steps of this systematic review and meta-analysis were based on the Meta-analyses Of Observational Studies in Epidemiology (MOOSE) protocol [42], and the results were reported accordance with the Preferred Reporting Items for Systematic Reviews and Meta-analysis (PRISMA) guideline [43]. Two authors independently preformed all study steps. In the case of dispute, a third author was involved. We registered this review at PROSPERO (registration number: CRD42020142861), Available from: https://www.crd.york.ac.uk/prospero/display_record.php?ID=CRD42020142861.

### Search strategy

To find potentially relevant published articles, comprehensive search was done on international online databases Scopus, Science Direct, EMBASE, PubMed/Medline, CINAHL, EBSCO, Cochrane Library (Cochrane Database of Systematic Reviews - CDSR), Web of Science and national online databases Iranian Research Institute for Information Science and Technology (IranDoc) (https://irandoc.ac.ir), Scientific Information Database (SID) (http://www.sid.ir/), Magiran (http://www.magiran.com/), Regional Information Center for Science and Technology (RICST) (http://en.ricest.ac.ir/), Iranian National Library (http://www.nlai.ir/), and Barakat Knowledge Network System (http://health.barakatkns.com) and the Google Scholar search engine in June 2019. Our search was done to retrieve all literature related to GERD in Iran. The reference list of articles was reviewed to find the gray literature. The studies identified by our search strategies were entered into Endnote X7 (Thomson Reuters, Philadelphia, PA, USA) software.

The related articles were searched in PubMed using a combination of expressions and terms Medical Subject Heading (MeSH): “gastroesophageal reflux”[MeSH Terms] OR “gastroesophageal reflux disease” [Text Word] OR “heartburn”[MeSH Terms] AND “Iran”[MeSH Terms]. Search terms were combined using Boolean operators of “OR” or “AND”.

### Study selection

The two researchers independently reviewed the articles on the abovementioned databases. The third researcher examined the consistency between the data extracted by the two researchers, and the contradictory results were discussed and resolved. After collecting literature from the databases, the next step was to assess whether the articles corresponded to the content of the title and abstract. The second and third stages were the review of the remaining articles with full text.

### Inclusion and exclusion criteria

We included the studies that were: (1) written in English or Persian; (2) cross-sectional studies; (3) with the primary aim of reporting the prevalence of GERD, heartburn and regurgitation; and (4) preformed among adults.

We excluded studies that: (1) had non-random sample size; (2) were non-relevant; (3) GERD diagnosis was not defined by heartburn and regurgitation; (4) were non-Iranian; (5) were case reports, review articles, congresses, letters to the editor without quantitative data, and theses.

### Data extraction and management

In case of duplicate publication, we contacted the researchers to clarify the original publication, and if we did not get an answer, we chose the study with the largest number of participants for cases with overlapping data, if necessary, additional details were extracted from the secondary articles.

We extracted the following data from each study: First author, year of publication, year of study, place of study, study design, method of diagnosis, data collection, characteristics of participants and estimation of prevalence.

### Qualitative assessment

The modified Newcastle Ottawa Scale (NOS) was used to assess the quality of studies [44]. The studies were divided into three categories based on the scores: high risk studies (scores ranging from 1 to 4), moderate risk (scores ranging from 5 to 7), and low risk (scores ranging from 8 to 10). Low and medium risk studies were included in the meta-analysis.

### Statistical analysis

The prevalence of the GERD is shown using the event rate. The 95% confidence intervals (CI) were calculated using Comprehensive Meta-Analysis (CMA) software ver 2 using sample size (N) and standard error (SE). To determine women to men ratio, we calculated the odds ratio (OR). Cochran Q test and I^2^ index were used to assess the heterogeneity of the studies. There are three categories for I^2^ index: I^2^ index below 25% is low heterogeneity, 25–75% is medium, and above 75% is high heterogeneity [45, 46]. For cases with low heterogeneity, the fixed effects model was used and for cases with medium and high heterogeneity, the random effects model was used. Subgroup analysis was used to find the cause of heterogeneity in the studies. Sensitivity analysis was performed by removing a study at a time to assess the predictive power. Mixed-effects meta-regression was used to investigate the relationship between continuous variables such as the time of study and the prevalence [47]. Finally, distribution bias was evaluated using funnel plot, and Egger and Begg’s tests. Statistical analysis and graph diagrams were performed using CMA version 2. The significance level of the test was considered to be *P* <  0.05.

## Results

### Search results and characteristics

**Fig. 1:**
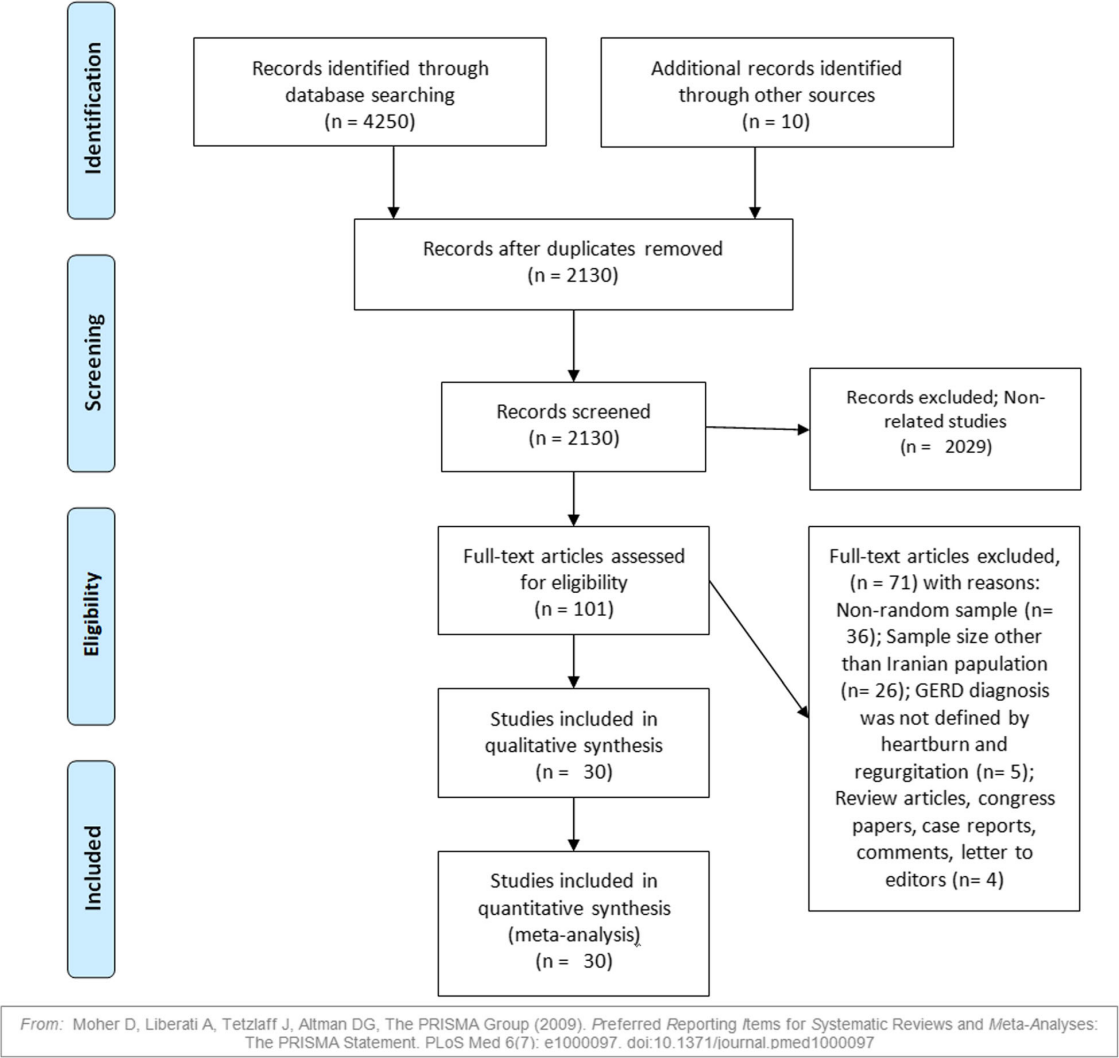
PRISMA process

Our initial search found 4260 records. After removing 2130 duplicates, 2130 unique documents were reviewed for relating the titles and abstract. Then, we reviewed the full text of 101 articles. Finally, 30 articles (23 studies for GERD, 20 studies for heartburn, and 13 studies for regurgitation) were included in the study (Fig. 1). The mean age of the participants (in 14 reported studies) was 39.35 years (95% CI: 34.98–43.71). Table 1 shows the characteristics of each study.

**Table 1:** Summary of characteristics in studies into a meta-analysis

Ref.	First author, Published Year	Year	Place	Population	Mean Age (±SD)	Method	Duration	Sample size			Quality
								All	Male	Female	
[15]	Nouraie et al., 2007	2005	Tehran	General population	36.1 ± 12.4	Questionnaire + Interview	NR	1202	505	697	Medium risk
[16]	Hatami et al., 2003	2001	Tehran	Blood Donors	37.22 ± 0.19	Questionnaire + Interview	12 M	3517	3115	402	Medium risk
[17]	Rogha et al., 2006	2004	Isfahan	General population	38.8 ± 12.9	Interview	12 M	2400	1074	1326	Medium risk
[18]	Mahmoudi et al., 2012	2001	Tehran	Medical students		Questionnaire + Interview	12 M	3008	1223	1785	Medium risk
[48]	Ehsani et al., 2007	1991	Tehran	General population		Questionnaire + Interview	NR	700	350	350	Low risk
[10]	Mostaghni et al., 2009	2006	Fars	Qashqai migrating nomad	43.1 ± 14.2	Questionnaire + Interview	12 M	717	284	433	Low risk
[32]	Aletaha et al., 2010	2005–6	Gonbad Kavoos, Kalale	General population	27.35 ± 6.1	Interview	12 M	1000			Medium risk
[33]	Nasseri- Moghaddam et al., 2008	2006	Tehran	General population	34.8 ± 13.0	Questionnaire + Interview	12 M	2057		1132	Low risk
[34]	Solhpour et al., 2008	2006	Damavand, Firoozkouh	General population	37.9 ± 14.3	Questionnaire + Interview	3 M	5733	2935	2798	Medium risk
[15]	Nouraie et al., 2007	2005	Tehran	General population		Questionnaire + Interview	6 M	2561	1083	1478	Medium risk
[35]	Saberi et al., 2010	2008–9	Kashan	Shift working nurses		Questionnaire	4 W	160			Low risk
[31]	Saberi-Firoozi M et al., 2007	2004	Shiraz	General population	49.9 ± 11.14	Questionnaire + Interview	12 M	1978	582	1396	Low risk
[19]	Somi et al., 2006	2005	Tabriz	Medical sciences studen	22.48 ± 1.98	Questionnaire + Interview	12 M	589			Medium risk
[36]	Hoseini-assal et al., 2004	2002	Shahrekord	General population	37.9 ± 14.3	Interview	12 M	4762	2045	2717	Medium risk
[20]	Pourshams et al., 2005	2002	Gonabad	General population		Interview	12 M	1066	450	616	Low risk
[21]	Bordbar et al., 2015	2013	Bandar Abbas	medical sciences students		Questionnaire	12 M	600	220	380	Medium risk
[37]	Vakhshoori et al., 2018	2010–12	Isfahan	Staff of Isfahan University of Medical Sciences	36.53	Questionnaire	3 M	4669			Low risk
[11]	Vossoughinia et al., 2014	2010	Mashhad	General population		Questionnaire	NR	1685			Low risk
[27]	Shahravan et al., 2013	2003	Sari	General population	38.4	Questionnaire	12 M	901	433	468	Medium risk
[22]	Pourhoseingholi et al., 2012	2006–7	Tehran	General population	38.7 ± 17.1	Questionnaire + Interview	3 M	18,180	9108	9072	Low risk
[38]	Mansour-Ghanaei et al., 2013	2010	Rasht	General population	38.31 ± 13.09	Questionnaire + Interview	NR	1473	453	1020	Low risk
[30]	Khodamoradi et al., 2017	2010	Fars	General population	52.6 ± 9.7	Questionnaire + Interview	12 M	9264	4276	4988	Low risk
[39]	Islami et al., 2014	2004–8	Golestan	General population	36.1 ± 12.4	Questionnaire + Interview	12 M	49,975	21,216	28,785	Low risk

*SD* standard deviation, *NR* not reported

### GERD prevalence and sensitivity analysis

**Fig. 2:**
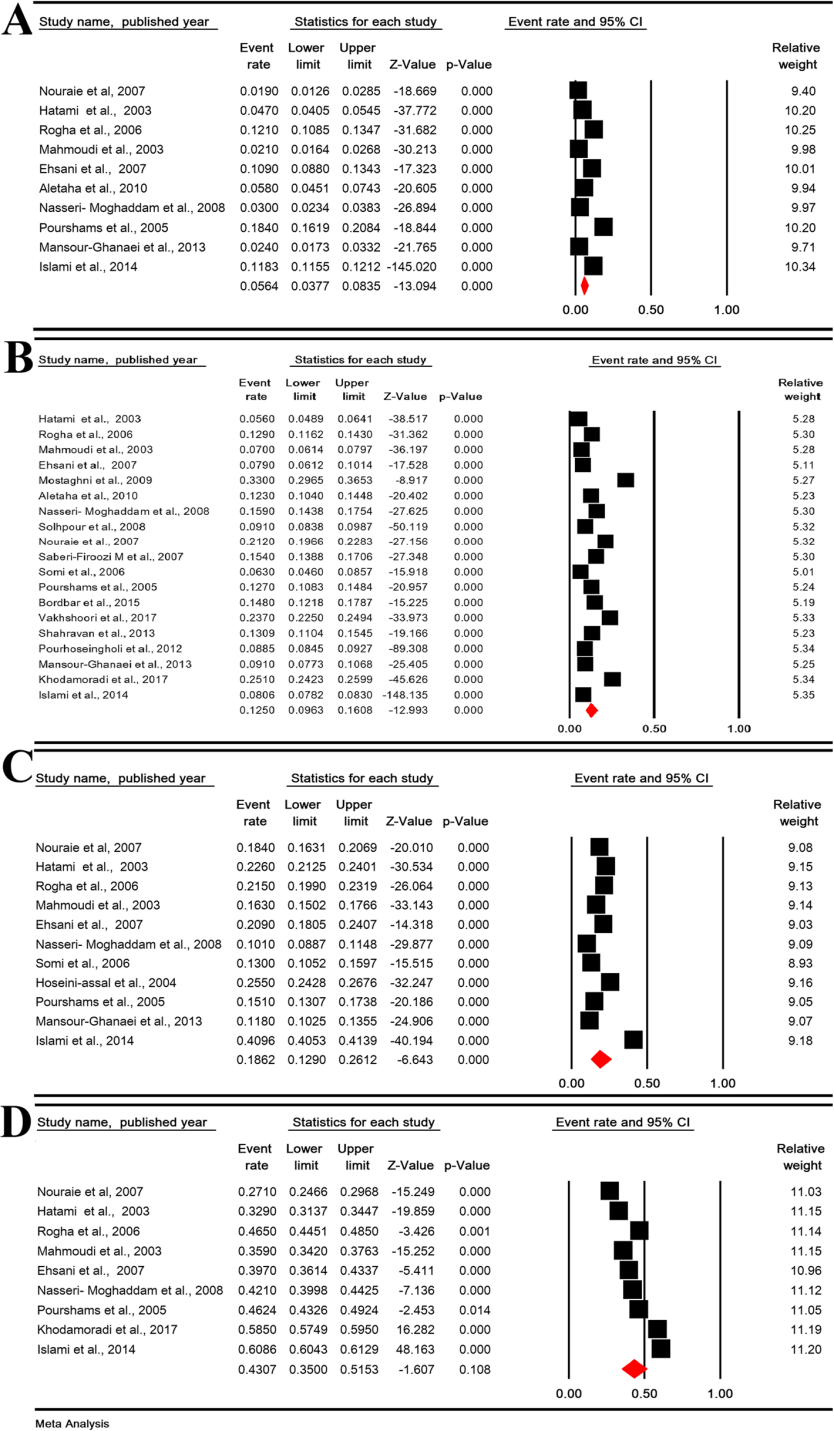
The daily (**a**), weekly (**b**), monthly (**c**), and overall (**d**) prevalence of GERD symptoms in Iranian population

The daily, weekly, monthly, and overall prevalence of GERD symptoms in Iranian population was 5.64% (95% CI: 3.77–8.35%; heterogeneity: I^2^ = 98.76%, *P* <  0.001; *N* = 66,398), 12.50% (95% CI: 9.63–16.08%; heterogeneity: I^2^ = 99.50%, *P* <  0.001; *N* = 110,388), 18.62% (95% CI: 12.90–26.12%; heterogeneity: I^2^ = 99.66%, *P* <  0.001; *N* = 70,749) and 43.07% (95% CI: 35.00–51.53%; heterogeneity: I^2^ = 99.66%, *P* <  0.001; *N* = 73,189), respectively (Fig. 2).

The sensitivity analysis for prevalence of all types GERD symptoms by removing a study showed that the overall estimate is still robust (Figure 1- supplementary).

### Subgroup analysis of GERD

The subgroup analysis for the daily, weekly, monthly, and overall prevalence of GERD symptoms is shown in Table 2. For the daily prevalence of GERD, the subgroup analysis of the study population (*P* <  0.001) and the data collection method (*P* = 0.019) were significant. For the weekly prevalence of GERD, subgroup analysis of the area (*P* = 0.001) and study population (*P* <  0.001) were significant. For the monthly prevalence of GERD, the subgroup analysis of the study population was significant (*P* = 0.001). For the overall prevalence of GERD, the subgroup analysis of the area (*P* <  0.001), the study population (*P* <  0.001) and the quality of studies (*P* = 0.005) were significant. Other variables were not significant.

**Table 2:** Subgroup analysis of prevalence of GERD

Variable			Studies (N)	Sample (N)		Heterogeneity		95% CI	Pooled prevalence (%)
				Total subjects	Event	I^2^	*P*-Value		
**Daily**	**Areas**	Center	6	12,884	680	98.44	< 0.001	2.37–8.47	4.52
		East	2	2066	254	98.54	< 0.001	3.21–29.70	10.58
		North	2	51,448	5947	98.98	< 0.001	1.09–23.40	5.48
		Test for subgroup differences: Q = 1.559, df(Q) = 2, *P* = 0.459							
	**Population**	Blood donors	1	3517	165	–	–	4.05–5.45	4.70
		General population	8	59,873	6653	98.18	< 0.001	4.51–9.45	6.56
		Health care worker	1	3008	63	–	–	1.64–2.68	2.10
		Test for subgroup differences: Q = 38.389, df(Q) = 2, *P* < 0.001							
	**Year of studies**	1991–2004	6	11,691	849	98.65	< 0.001	4.03–13.07	7.37
		2005–2013	4	54,707	6032	99.01	< 0.001	1.20–10.51	3.64
		Test for subgroup differences: Q = 1.256, df(Q) = 1, *P* = 0.263							
	**Quality of studies**	Low risk	5	55,271	6282	98.52	< 0.001	4.21–12.39	7.31
		Moderate risk	5	11,127	600	98.46	< 0.001	2.08–8.54	4.26
		Test for subgroup differences: Q = 1.380, df(Q) = 1, *P* = 0.240							
	**Method of data collection**	Questionnaire + Interview	7	61,932	6337	99.06	< 0.001	2.14–7.81	4.12
		Interview	3	4466	545	98.91	< 0.001	6.53–18.38	11.14
		Test for subgroup differences: Q = 5.488, df(Q) = 1, *P* = 0.019							
	**Sex**	The odds ratio of females to males: 1.503 (95% CI: 1.153–1.59, *P* = 0.003); Heterogeneity: I^2^: 68.49%, *P* = 0.013							
**Weekly**	**Areas**	Center	9	42,825	4880	99.34	< 0.001	7.92–15.92	11.31
		East	2	2066	258	0	0.784	11.15–14.01	12.51
		North	4	52,938	4317	91.08	< 0.001	7.04–11.38	8.98
		South	4	12,559	2955	97.89	< 0.001	15.22–28.89	21.26
		Test for subgroup differences: Q = 17.025, df(Q) = 3, *P* = 0.001							
	**Population**	Blood donors	1	3517	197	–	–	4.89–6.41	5.60
		General population	14	98,005	10,770	99.69	< 0.001	10.07–17.91	13.52
		Health care worker	4	8866	1443	99.20	< 0.001	5.17–7.39	11.44
		Test for subgroup differences: Q = 29.288, df(Q) = 2, *P* < 0.001							
	**Year of studies**	1991–2004	8	14,570	1453	97.25	< 0.001	7.86–13.56	10.37
		2005–2013	11	95,818	10,957	99.70	< 0.001	9.95–20.04	14.27
		Test for subgroup differences: Q = 1.947, df(Q) = 1, *P* = 0.163							
	**Quality of studies**	Low risk	10	90,079	10,262	99.71	< 0.001	9.85–20.74	14.47
		Moderate risk	9	20,309	2149	98.26	< 0.001	7.65–14.46	10.58
		Test for subgroup differences: Q = 1.544, df(Q) = 1, *P* = 0.214							
	**Method of data collection**	Interview	3	4466	568	0	0.892	11.77–13.73	12.72
		Questionnaire	3	6170	1313	96.95	< 0.001	10.71–25.45	16.83
		Questionnaire + Interview	13	99,752	10,529	99.61	< 0.001	8.38–15.92	11.63
		Test for subgroup differences: Q = 1.815, df(Q) = 2, *P* = 0.404							
	**Sex**	The odds ratio of females to males: 1.174 (95% CI: 0.974–1.414, *P* = 0.092); Heterogeneity: I^2^: 91.63%, *P* < 0.001							
**Monthly**	**Areas**	Center	7	17,646	3591	97.55	< 0.001	15.36–22.91	18.84
		East	1	1066	161	–	–	13.86–16.42	15.10
		North	3	52,037	20,720	99.64	< 0.001	6.22–46.66	19.42
		Test for subgroup differences: Q = 3.177, df(Q) = 2, *P* = 0.204							
	**Population**	Blood donors	1	3517	795	98.91	< 0.001	21.25–24.01	22.60
		General population	8	63,635	23,110	99.71	< 0.001	12.44–28.62	19.27
		Health care worker	2	3597	567	98.23	< 0.001	11.92–18.40	14.87
		Test for subgroup differences: Q = 14.531, df(Q) = 2, *P* = 0.001							
	**Year of studies**	1991–2004	6	15,453	3323	95.89	< 0.001	17.14–23.54	20.15
		2005–2013	5	55,296	21,149	99.70	< 0.001	7.27–34.71	16.95
		Test for subgroup differences: Q = 0.181, df(Q) = 1, *P* = 0.671							
	**Quality of studies**	Low risk	5	55,271	21,159	99.70	< 0.001	7.82–35.92	17.90
		Moderate risk	6	15,478	3313	96.03	< 0.001	16.42–22.85	19.43
		Test for subgroup differences: Q = 0.042, df(Q) = 1, *P* = 0.838							
	**Method of data collection**	Interview	3	8228	1891	97.45	< 0.001	15.89–26.03	20.50
		Questionnaire + Interview	8	62,521	22,581	99.70	< 0.001	10.79–28.45	17.99
		Test for subgroup differences: Q = 0.233, df(Q) = 1, *P* = 0.637							
	**Sex**	The odds ratio of females to males: 1.126 (95% CI: 0.849–1.494, *P* = 0.411); Heterogeneity: I^2^: 96.68%, *P* < 0.001							
**Overall**	**Areas**	Center	6	12,884	4823	97.38	< 0.001	32.01–42.62	37.16
		East	1	1066	493	–	–	43.26–49.24	46.24
		North	1	49,975	30,415	–	–	60.43–61.26	60.86
		South	1	9264	5419	–	–	57.49–59.50	58.50
		Test for subgroup differences: Q = 169.751, df(Q) = 3, *P* < 0.001							
	**Population**	Blood donors	1	3517	1157	–	–	31.37–34.47	32.90
		General population	7	66,664	38,913	99.43	< 0.001	38.49–53.12	45.71
		Health care worker	1	3008	1080	99.09	< 0.001	34.20–37.63	35.90
		Test for subgroup differences: Q = 16.155, df(Q) = 2, *P* < 0.001							
	**Year of studies**	1991–2004	5	10,691	4124	97.26	< 0.001	34.36–46.09	40.09
		2005–2013	4	62,498	37,026	99.59	< 0.001	37.71–56.28	46.89
		Test for subgroup differences: Q = 1.458, df(Q) = 1, *P* = 0.227							
	**Quality of studies**	Low risk	5	63,062	37,471	99.15	< 0.001	43.12–56.23	49.67
		Moderate risk	4	10,127	3679	98.20	< 0.001	28.59–42.77	35.36
		Test for subgroup differences: Q = 8.008, df(Q) = 1, *P* = 0.005							
	**Method of data collection**	Questionnaire + Interview	7	69,723	39,541	99.73	< 0.001	32.71–52.17	42.14
		Interview	2	3466	1609	0	< 0.001	44.76–48.08	46.42
		Test for subgroup differences: Q = 0.692, df(Q) = 1, *P* = 0.406							
	**Sex**	The odds ratio of females to males: 1.111 (95% CI: 0.888–1.391, *P* = 0.358); Heterogeneity: I^2^: 97.96%, *P* < 0.001							

*CI* Confidence intervals, *N* number

### The prevalence of GERD by gender

The daily, weekly, monthly, and overall prevalence of GERD symptoms in Iranian males was 5.72% (95% CI: 3.41–9.46%; heterogeneity: I^2^ = 97.44%, *P* <  0.001; *N* = 26,004), 11.38% (95% CI: 8.10–15.75%; heterogeneity: I^2^ = 97.80%, *P* <  0.001; *N* = 19,453), 15.68% (95% CI: 10.67–22.45%; heterogeneity: I^2^ = 98.15%, *P* <  0.001; *N* = 8865) and 39.26% (95% CI: 32.35–46.62%; heterogeneity: I^2^ = 99.04%, *P* <  0.001; *N* = 31,704) (Figure 2-supplementary).

**Fig. 3:**
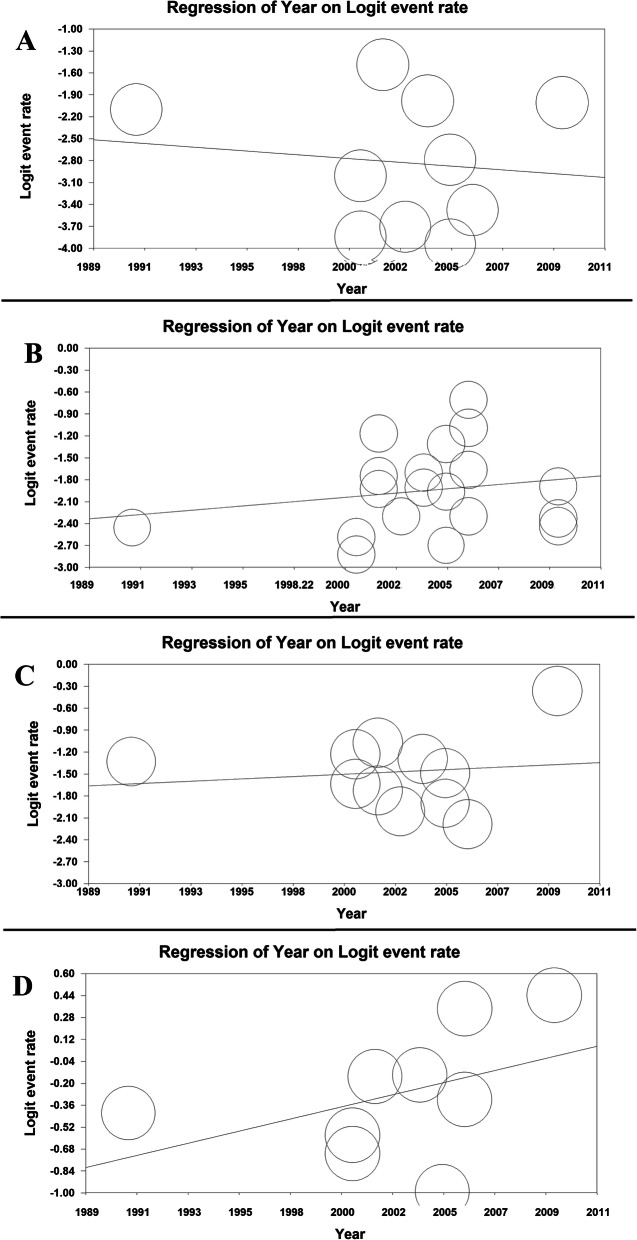
The meta-regression model based on years of study for daily (**a**), weekly (**b**), monthly (**c**), and overall (**d**) prevalence of GERD

The daily, weekly, monthly, and overall prevalence of GERD symptoms in Iranian females was 7.88% (95% CI: 3.67–16.11%; heterogeneity: I^2^ = 98.56%, *P* <  0.001; *N* = 31,588), 12.81% (95% CI: 9.47–17.10%; heterogeneity: I^2^ = 98.04%, *P* <  0.001; *N* = 19,380), 16.96% (95% CI: 13.17–21.56%; heterogeneity: I^2^ = 98.17%, *P* <  0.001; *N* = 21,567), and 45.51% (95% CI: 38.22–52.99%; heterogeneity: I^2^ = 98.99%, *P* <  0.001; *N* = 38,252) (Figure 3-supplementary).

Odds ratio (OR) for the prevalence of daily, weekly, monthly, and overall prevalence of GERD in women compared to men in Table 2 shows that there is a significant difference only in the daily prevalence of GERD (*P* = 0.003).

### Meta-regression and publication bias for prevalence of GERD

The meta-regression model based on years of study for GERD prevalence revealed that the meta-regression coefficient for daily, weekly, monthly, and overall prevalence of GERD was (− 0.022, 95% CI: − 0.132 to 0.087, *P*= 0.688), (0.025, 95% CI: − 0.410 to 0.092, *P*= 0.450), (0.0140, 95% CI: − 0.057 to 0.085, *P* = 0.700) and (0.038, 95% CI: − 0.081 to 0.085, *P*= 0.104), respectively (Fig. 3).

**Fig. 4:**
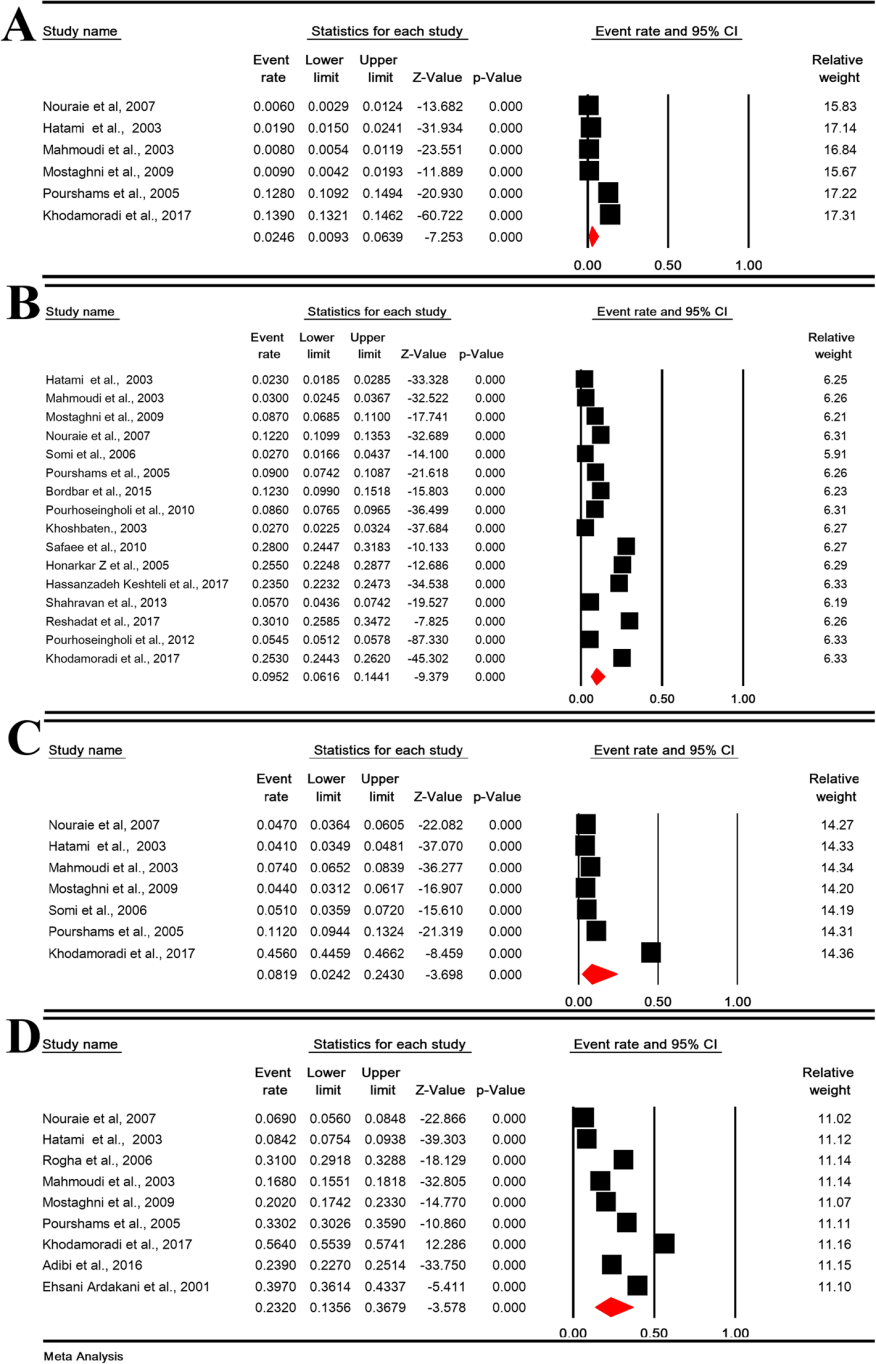
The daily (**a**), weekly (**b**), monthly (**c**), and overall (**d**) prevalence of heartburn in Iranian population

Regarding publication bias, the significance level of Egger and Begg’s tests was (Egger = 0.024 and Begg’s = 0.152), (Egger = 0.628 and Begg’s = 0.624), (Egger< 0.001 and Begg’s = 0.533) and (Egger = 0.002 and Begg’s = 0.754) for the daily, weekly, monthly, and overall prevalence of GERD, respectively (Figure 4-supplementary).

### Heartburn prevalence and sensitivity analysis

The daily, weekly, monthly, and overall prevalence of heartburn in Iranian population was 2.46% (95% CI: 0.93–6.39%; heterogeneity: I^2^ = 99.15%, *P* <  0.001; *N* = 18,774), 9.52% (95% CI: 6.16–14.41%; heterogeneity: I^2^ = 99.58%, *P* <  0.001; *N* = 54,125), 8.19% (95% CI: 2.42–24.30%; heterogeneity: I^2^ = 99.76%, *P* <  0.001; *N* = 19,363) and 23.20% (95% CI: 13.56–36.79%; heterogeneity: I^2^ = 99.77%, *P* <  0.001; *N* = 26,543), respectively (Fig. 4).

**Fig. 5:**
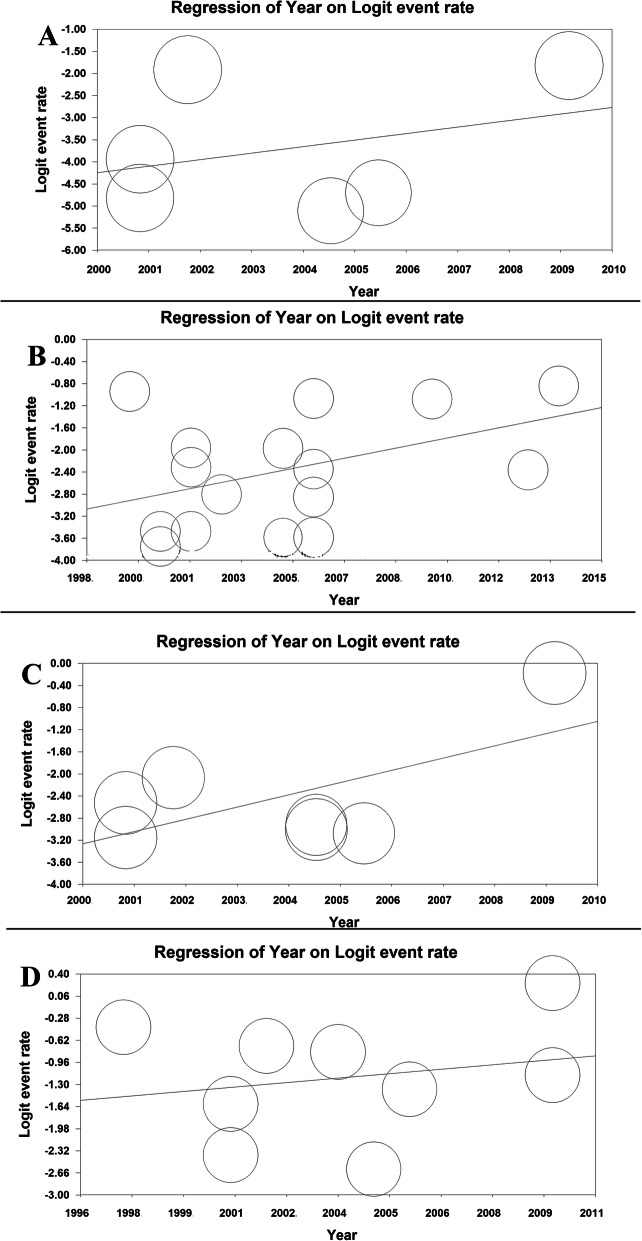
The meta-regression model based on years of study for daily (**a**), weekly (**b**), monthly (**c**), and overall (**d**) prevalence of heartburn

The sensitivity analysis for prevalence of all types heartburn symptoms by removing a study showed that the overall estimate is still robust (Figure 5-Supplement).

### Subgroup analysis of heartburn

For the daily prevalence of heartburn, the subgroup analysis of the area (*P* <  0.001), study population (*P* <  0.001), the quality of studies (*P* <  0.001) and method of data collection (*P* = 0.007) were significant (Table 3). For the weekly prevalence of heartburn, subgroup analysis of the area (*P* = 0.001), study population (*P* <  0.001) and year of study (*P* = 0.021) were significant (Table 3). For the monthly prevalence of heartburn, the subgroup analysis of the area (*P* <  0.001) and population (*P* = 0.044) was significant (Table 3). For the overall prevalence of heartburn, the subgroup analysis of the area (*P* = 0.019), and the study population (*P* <  0.001) were significant (Table 3). Other variables were not significant.

**Table 3:** Subgroup analysis of prevalence of heartburn

Variable			Studies (N)	Sample (N)		Heterogeneity		95% CI	Pooled prevalence (%)
				Total subjects	Event	I^2^	*P*-Value		
**Daily**	**Areas**	Center	3	7727	98	89.58	< 0.001	0.48–2.13	1.02
		East	1	1066	136	–	–	10.92–14.94	12.80
		South	2	9981	1294	98.10	< 0.001	0.23–39.75	3.78
		Test for subgroup differences: Q = 46.616, df(Q) = 2, *P* < 0.001							
	**Population**	Blood donors	1	3517	67	–	–	1.50–2.41	1.90
		General population	4	12,249	1438	97.67	< 0.001	1.86–7.92	3.88
		Health care worker	1	3008	24	–	–	0.54–1.19	0.80
		Test for subgroup differences: Q = 19.304, df(Q) = 2, *P* < 0.001							
	**Year of studies**	1998–2005	4	8793	235	98.02	< 0.001	0.42–8.35	1.93
		2006–2015	2	9981	1294	98.10	< 0.001	0.23–39.75	3.78
		Test for subgroup differences: Q = 0.672, df(Q) = 1, *P* = 0.672							
	**Quality of studies**	Low risk	3	11,047	1431	98.84	< 0.001	4.27–12.53	7.40
		Moderate risk	3	7727	98	89.58	< 0.001	0.48–2.13	1.02
		Test for subgroup differences: Q = 17.950, df(Q) = 1, *P* < 0.001							
	**Method of data collection**	Questionnaire + Interview	5	17,708	1392	99.31	< 0.001	0.37–7.43	1.69
		Interview	1	1066	136	–	–	10.92–14.94	12.80
		Test for subgroup differences: Q = 7.342, df(Q) = 1, *P* = 0.007							
	**Sex**	The odds ratio of females to males: 1.211 (95% CI: 0.915–1.602, *P* = 0.180); Heterogeneity: I^2^: 0%, *P* = 0.829							
**Weekly**	**Areas**	Center	7	35,634	3014	99.66	< 0.001	4.38–16.29	8.62
		East	1	1066	96	–	–	7.42–10.87	9.00
		North	3	5697	181	90.56	< 0.001	2.04–5.97	3.50
		South	4	11,318	2668	97.75	< 0.001	10.64–25.31	16.37
		West	1	410	123	–	–	25.85–34.72	30.10
		Test for subgroup differences: Q = 131.724, df(Q) = 4, *P* < 0.001							
	**Population**	Blood donors	1	3517	81	–	–	1.85–2.85	2.30
		General population	11	45,674	5633	99.65	< 0.001	7.14–18.48	11.66
		Health care worker	3	4197	180	97.84	< 0.001	1.60–13.25	4.74
		injured people of B	1	737	188	–	–	22.48–28.77	25.50
		Test for subgroup differences: Q = 364.779, df(Q) = 3, *P* < 0.001							
	**Year of studies**	1991–2004	8	16,586	948	99.03	< 0.001	2.86–10.91	5.66
		2005–2013	8	37,539	5133	99.70	< 0.001	8.94–25.47	15.48
		Test for subgroup differences: Q = 5.330, df(Q) = 1, *P* = 0.021							
	**Quality of studies**	Low risk	6	32,832	3913	99.76	< 0.001	5.58–24.24	12.08
		Moderate risk	10	21,296	2169	99.39	< 0.001	4.36–14.88	8.19
		Test for subgroup differences: Q = 0.614, df(Q) = 1, *P* = 0.433							
	**Method of data collection**	Interview	2	4100	357	0	0.690	7.88–9.61	8.71
		Questionnaire	4	7001	1432	98.09	< 0.001	8.76–24.18	14.90
		Questionnaire + Interview	10	43,024	4292	99.70	< 0.001	4.11–15.09	8.03
		Test for subgroup differences: Q = 3.897, df(Q) = 2, *P* = 0.142							
	**Sex**	The odds ratio of females to males: 1.678 (95% CI: 1.105–2.548, *P* = 0.015); Heterogeneity: I^2^: 80.16%, *P* < 0.001							
**Monthly**	**Areas**	Center	3	7727	423	94.26	< 0.001	3.46–7.91	5.26
		East	1	1066	119	–	–	9.44–13.24	11.20
		North	1	589	30	–	–	3.59–7.20	5.10
		South	2	9981	4256	99.60	< 0.001	1.14–77.24	16.49
		Test for subgroup differences: Q = 27.0761, df(Q) = 3, *P* < 0.001							
	**Population**	Blood donors	1	3517	144	–	–	3.49–4.81	4.10
		General population	4	12,249	4432	99.69	< 0.001	2.40–38.88	11.11
		Health care worker	2	3597	253	74.63	< 0.001	4.44–9.07	6.37
		Test for subgroup differences: Q = 6.229, df(Q) = 2, *P* = 0.044							
	**Year of studies**	1991–2004	5	9382	573	95.15	< 0.001	4.16–8.93	6.12
		2005–2013	2	9981	4256	99.60	< 0.001	1.14–77.24	16.49
		Test for subgroup differences: Q = 0.571, df(Q) = 1, *P* = 0.450							
	**Quality of studies**	Low risk	3	11,047	4375	99.66	< 0.001	2.96–48.85	14.57
		Moderate risk	4	8316	453	91.48	< 0.001	3.71–7.31	5.23
		Test for subgroup differences: Q = 1.582, df(Q) = 1, *P* = 0.208							
	**Method of data collection**	Interview	1	1066	119	–	–	9.44–13.24	11.20
		Questionnaire + Interview	6	18,297	4709	99.81	< 0.001	1.90–26.74	7.76
		Test for subgroup differences: Q = 0.288, df(Q) = 1, *P* = 0.592							
	**Sex**	The odds ratio of females to males: 1.282 (95% CI: 1.282–1.729, *P* < 0.001); Heterogeneity: I^2^: 16.13%, *P* = 0.311							
**Overall**	**Areas**	Center	6	15,496	3022	99.35	< 0.001	11.70–27.69	18.38
		East	1	1066	352	–	–	30.26–35.90	33.02
		South	2	9981	5370	99.65	< 0.001	10.39–73.94	36.45
		Test for subgroup differences: Q = 7.973, df(Q) = 2, *P* = 0.019							
	**Population**	Blood donors	1	3517	369	–	–	7.54–9.38	8.42
		General population	6	15,349	6827	99.62	< 0.001	16.36–44.01	28.17
		Health care worker	2	7677	1621	98.17	< 0.001	14.06–27.99	20.14
		Test for subgroup differences: Q = 34.143, df(Q) = 2, *P* < 0.001							
	**Year of studies**	1991–2004	6	11,893	2258	99.39	< 0.001	11.40–31.36	19.52
		2005–2013	3	14,650	6486	99.85	< 0.001	13.15–59.27	31.94
		Test for subgroup differences: Q = 0.996, df(Q) = 1, *P* = 0.318							
	**Quality of studies**	Low risk	4	15,716	6838	99.83	< 0.001	16.21–53.86	32.22
		Moderate risk	5	10,827	1906	99.45	< 0.001	9.22–30.35	17.38
		Test for subgroup differences: Q = 1.908, df(Q) = 1, *P* = 0.167							
	**Method of data collection**	Interview	2	3466	1096	99.44	< 0.001	29.86–33.66	31.73
		Questionnaire	2	5369	1394	98.69	< 0.001	18.00–48.35	31.19
		Questionnaire + Interview	5	17,708	6254	99.87	< 0.001	5.54–49.93	17.66
		Test for subgroup differences: Q = 1.148, df(Q) = 2, *P* = 0.505							
	**Sex**	The odds ratio of females to males: 1.414 (95% CI: 1.093–1.829, *P* = 0.008); Heterogeneity: I^2^: 79.84%, *P* = 0.002							

*CI* Confidence intervals, *N* number

### The prevalence of heartburn by gender

**Fig. 6:**
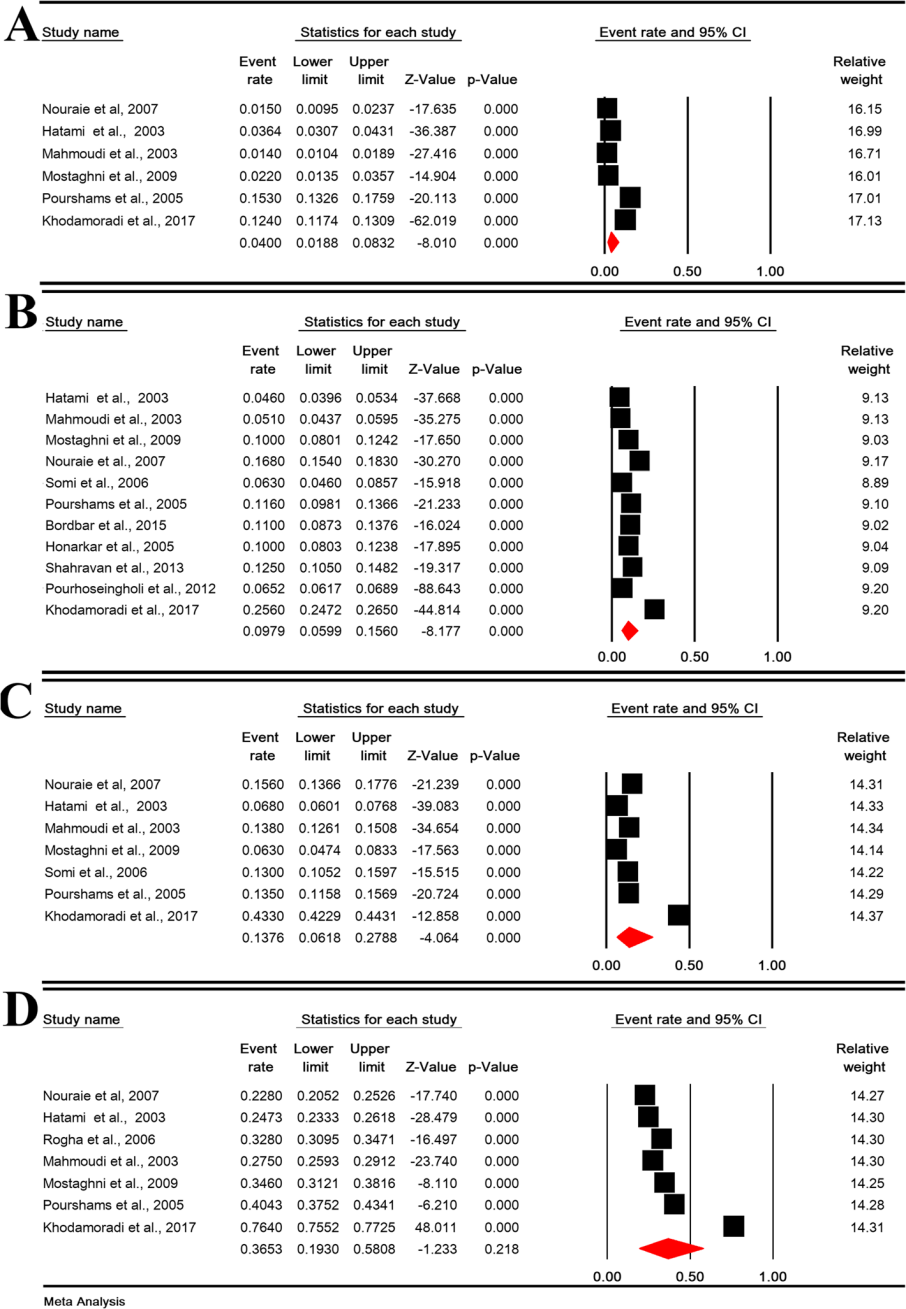
The daily (**a**), weekly (**b**), monthly (**c**), and overall (**d**) prevalence of regurgitation in Iranian population

The daily, weekly, monthly, and overall prevalence of heartburn in Iranian males was 2.61% (95% CI: 0.59–10.75%; heterogeneity: I^2^ = 98.19%, *P* <  0.001; *N* = 4778), 5.68% (95% CI: 1.81–16.44%; heterogeneity: I^2^ = 98.69%, *P* <  0.001; *N* = 7257), 5.93% (95% CI: 3.93–8.84%; heterogeneity: I^2^ = 89.65%, *P* <  0.001; *N* = 4788) and 16.54% (95% CI: 10.9–24.28%; heterogeneity: I^2^ = 96.43%, *P* <  0.001; *N* = 1788) (Figure 6-supplementary).

**Fig. 7:**
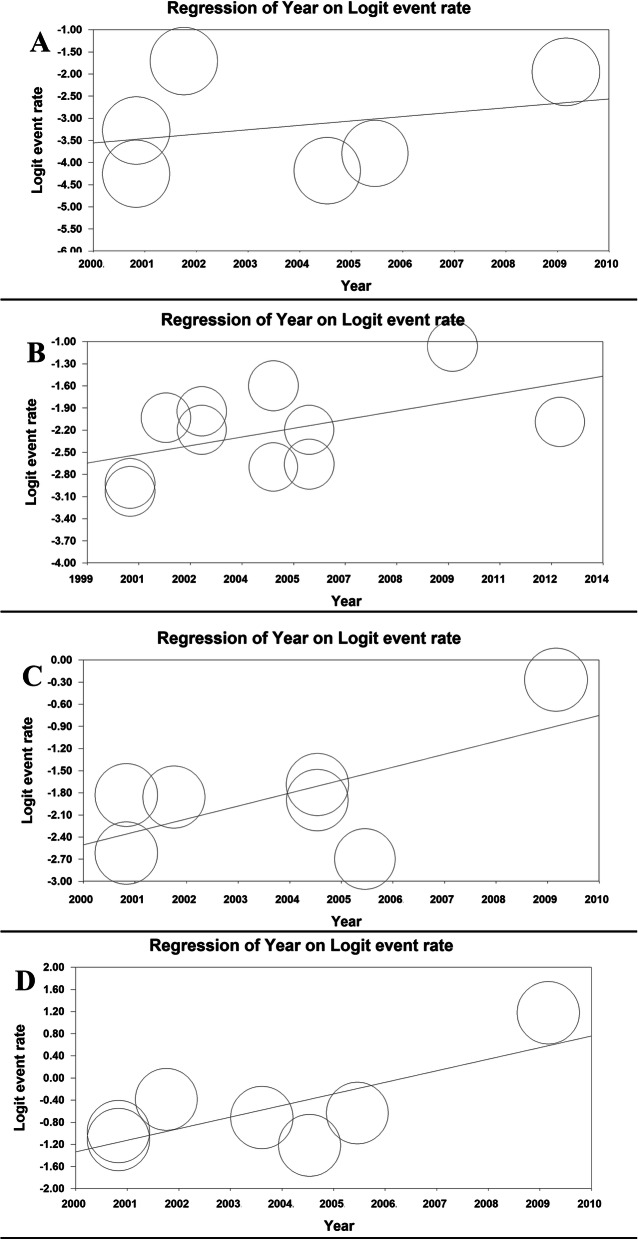
The meta-regression model based on years of study for daily (**a**), weekly (**b**), monthly (**c**), and overall (**d**) prevalence of regurgitation

The daily, weekly, monthly, and overall prevalence of heartburn in Iranian females was 2.90% (95% CI: 0.36–19.95%; heterogeneity: I^2^ = 98.45%, *P* <  0.001; *N* = 2803), 6.89% (95% CI: 2.96–15.21%; heterogeneity: I^2^ = 98.02%, *P* <  0.001; *N* = 5171), 9.90% (95% CI: 6.45–14.90%; heterogeneity: I^2^ = 92.19%, *P* <  0.001; *N* = 3183), 19.71% (95% CI: 11.89–30.89%; heterogeneity: I^2^ = 98.02%, *P* <  0.001; *N* = 2803) (Figure 7-supplementary).

OR for the prevalence of daily, weekly, monthly, and overall prevalence of heartburn in women compared to men in Table 3 shows that there is a significant difference in the weekly (*P* = 0.015), monthly (*P* <  0.001) and overall (*P* = 0.008) prevalence of heartburn.

### Meta-regression and publication bias for prevalence of heartburn

The meta-regression model based on years of study for heartburn prevalence revealed that the meta-regression coefficient for daily, weekly, monthly, and overall prevalence of heartburn was (0.136, 95% CI: − 0.241 to 0.514, *P*= 0.478), (0.109, 95% CI: 0.013 to 0.205, *P*= 0.025), (0.205, 95% CI: 0.004 to 0.405, *P* = 0.044) and (0.047, 95% CI: − 0.103 to 0.198, *P*= 0.539), respectively (Fig. 5).

Regarding publication bias, the significance level of Egger and Begg’s tests was (Egger = 0.028 and Begg’s = 0.707), (Egger = 0.118 and Begg’s = 0.392), (Egger = 0.005 and Begg’s = 0.548) and (Egger = 0.025 and Begg’s = 0.754) for the daily, weekly, monthly, and overall prevalence of heartburn, respectively (Figure 8-supplementary).

### Regurgitation prevalence and sensitivity analysis

The daily, weekly, monthly, and overall prevalence of regurgitation in Iranian population was 4.00% (95% CI: 1.88–8.32%; heterogeneity: I^2^ = 99.03%, *P* <  0.001; *N* = 18,774), 9.79% (95% CI: 5.99–15.60%; heterogeneity: I^2^ = 99.55%, *P* <  0.001; *N* = 41,140), 13.76% (95% CI: 6.18–27.88%; heterogeneity: I^2^ = 99.73%, *P* <  0.001; *N* = 19,363) and 36.53% (95% CI: 19.30–58.08%; heterogeneity: I^2^ = 99.86%, *P* <  0.001; *N* = 21,174), respectively (Fig. 6).

The sensitivity analysis for prevalence of all types regurgitation symptoms by removing a study showed that the overall estimate is still robust (Figure 9-Supplement).

### Subgroup analysis of regurgitation

For the daily prevalence of regurgitation, the subgroup analysis of the area (*P* <  0.001), study population (*P* <  0.001), the quality of studies (*P* <  0.001) and the data collection method (*P* = 0.001) were significant (Table 4). For the weekly prevalence of regurgitation, subgroup analysis of the study population (*P* = 0.001) was significant (Table 4). For the monthly regurgitation of heartburn, the subgroup analysis of the population was significant (*P* <  0.001) (Table 4). For the overall prevalence of regurgitation, the subgroup analysis of the area (*P* < 0.001) was significant (Table 4). Other variables were not significant.

**Table 4:** Subgroup analysis of prevalence of regurgitation

Variable			Studies (N)	Sample (N)		Heterogeneity		95% CI	Pooled prevalence (%)
				Total subjects	Event	I^2^	*P*-Value		
**Daily**	**Areas**	Center	3	7727	188	94.21	< 0.001	0.97–4.09	2.00
		East	1	1066	163	–	–	13.26–17.59	15.30
		South	2	9981	1165	98.05	< 0.001	00.94–25.82	5.43
		Test for subgroup differences: Q = 33.289, df(Q) = 2, *P* < 0.001							
	**Population**	Blood donors	1	3517	128	–	–	3.07–4.31	3.64
		General population	4	12,249	1346	97.96	< 0.001	2.78–10.41	5.45
		Health care worker	1	3008	42	–	–	1.04–1.89	1.40
		Test for subgroup differences: Q = 33.09, df(Q) = 2, *P* < 0.001							
	**Year of studies**	1998–2005	4	8793	351	99.04	< 0.001	1.04–10.53	3.40
		2006–2015	2	9981	1162	98.05	< 0.001	0.94–25.82	5.43
		Test for subgroup differences: Q = 0.196, df(Q) = 1, *P* = 0.658							
	**Quality of studies**	Low risk	3	11,047	1328	98.90	< 0.001	5.03–13.76	8.42
		Moderate risk	3	7727	188	94.56	< 0.001	0.97–4.09	2.00
		Test for subgroup differences: Q = 10.268, df(Q) = 1, *P* < 0.001							
	**Method of data collection** **Sex**	Questionnaire + Interview	5	17,708	1353	99.17	< 0.001	1.07–8.02	2.98
		Interview	1	1066	163	99.51	< 0.001	13.26–17.59	15.30
		Test for subgroup differences: Q = 10.819, df(Q) = 1, *P* = 0.001							
		The odds ratio of females to males: 1.315 (95% CI: 0.786–2.201, *P* = 0.297); Heterogeneity: I^2^: 64.23%, *P* = 0.061							
**Weekly**	**Areas**	Center	4	27,266	1931	99.22	< 0.001	4.02–12.65	7.23
		East	1	1066	124	–	–	9.81–13.66	11.60
		North	2	1490	150	93.15	< 0.001	4.53–17.19	9.03
		South	4	11,318	2583	98.55	< 0.001	6.71–24.37	13.21
		Test for subgroup differences: Q = 3.130, df(Q) = 3, *P* = 0.372							
	**Population**	Blood donors	1	3517	162	–	–	3.96–5.34	4.60
		General population	6	32,689	4296	99.71	< 0.001	6.71–23.16	12.83
		Health care worker	3	4197	257	93.11	< 0.001	4.27–11.51	7.08
		injured people of B	1	737	74	–	–	8.03–12.38	1.00
		Test for subgroup differences: Q = 38.144, df(Q) = 3, *P* < 0.001							
	**Year of studies**	1991–2004	7	12,379	1093	98.18	< 0.001	5.55–13.53	8.75
		2005–2013	4	28,761	3695	99.82	< 0.001	4.51–27.80	11.89
		Test for subgroup differences: Q = 6.547, df(Q) = 1, *P* = 0.563							
	**Quality of studies**	Low risk	4	29,227	3753	99.83	< 0.001	4.68–27.62	12.04
		Moderate risk	7	11,913	1035	98.16	< 0.001	5.41–13.62	8.67
		Test for subgroup differences: Q = 0.393, df(Q) = 1, *P* = 0.531							
	**Method of data collection**	Interview	1	1066	124	–	–	9.81–13.66	11.60
		Questionnaire	3	2238	252	22.68	< 0.001	9.85–12.86	11.27
		Questionnaire + Interview	7	37,836	4412	99.73	< 0.001	4.61–16.96	9.04
		Test for subgroup differences: Q = 0.552, df(Q) = 2, *P* = 0.759							
	**Sex**	The odds ratio of females to males: 0.856 (95% CI: 0.509–1.4339, *P* = 0.558); Heterogeneity: I^2^: 84.17%, *P* < 0.001							
**Monthly**	**Areas**	Center	3	7727	842	98.17	< 0.001	6.94–18.29	11.44
		East	1	1066	144	–	–	11.58–15.69	13.50
		North	1	589	77	–	–	10.52–15.97	13.00
		South	2	9981	4056	99.59	< 0.001	2.06–71.12	18.55
		Test for subgroup differences: Q = 0.552, df(Q) = 3, *P* = 0.907							
	**Population**	Blood donors	1	3517	239	–	–	6.01–7.68	6.80
		General population	4	12,249	4388	99.61	< 0.001	6.03–37.74	16.47
		Health care worker	2	3597	492	0	0.605	12.59–14.83	13.67
		Test for subgroup differences: Q = 88.495, df(Q) = 2, *P* < 0.001							
	**Year of studies**	1991–2004	5	9382	1062	96.48	< 0.001	8.80–16.47	12.12
		2005–2013	2	9981	4056	99.59	< 0.001	2.06–71.12	18.55
		Test for subgroup differences: Q = 0.167, df(Q) = 1, *P* = 0.683							
	**Quality of studies**	Low risk	3	11,047	4200	99.62	< 0.001	4.44–46.54	16.75
		Moderate risk	4	8316	918	97.28	< 0.001	7.92–17.23	11.80
		Test for subgroup differences: Q = 0.273, df(Q) = 1, *P* = 0.601							
	**Method of data collection**	Interview	1	1066	144	–	–	11.58–15.69	13.50
		Questionnaire + Interview	6	18,297	4975	99.76	< 0.001	5.64–29.99	13.80
		Test for subgroup differences: Q = 0.002, df(Q) = 1, *P* = 0.960							
	**Sex**	The odds ratio of females to males: 0.500 (95% CI: 0.085–2.952, *P* = 0.859); Heterogeneity: I^2^: 98.30%, *P* < 0.001							
**Overall**	**Areas**	Center	4	10,127	2758	95.05	< 0.001	23.09–31.00	26.86
		East	1	1066	431	–	–	37.53–43.41	40.43
		South	2	9981	7326	99.79	< 0.001	18.17–88.55	56.72
		Test for subgroup differences: Q = 26.883, df(Q) = 2, *P* < 0.001							
	**Population**	Blood donors	1	3517	870	–	–	23.33–26.18	24.73
		General population	5	14,649	8818	99.84	< 0.001	19.28–67.23	41.18
		Health care worker	1	3008	827	–	–	25.93–29.12	27.50
		Test for subgroup differences: Q = 8.028, df(Q) = 2, *P* = 0.018							
	**Year of studies**	1991–2004	5	11,193	3198	97.12	< 0.001	24.40–34.70	29.28
		2005–2013	2	9981	7326	99.79	< 0.001	17.17–88.55	56.72
		Test for subgroup differences: Q = 1.587, df(Q) = 1, *P* = 0.208							
	**Quality of studies**	Low risk	3	11,047	7757	99.78	< 0.001	22.40–79.34	51.29
		Moderate risk	4	10,127	2758	95.02	< 0.001	23.09–31.00	26.86
		Test for subgroup differences: Q = 2.483, df(Q) = 1, *P* = 0.115							
	**Method of data collection**	Interview	2	3466	1218	94.67	< 0.001	29.35–44.21	36.46
		Questionnaire + Interview	5	17,708	9297	99.90	< 0.001	14.91–65.41	36.53
		Test for subgroup differences: Q = 0.000, df(Q) = 1, *P* = 0.996							
	**Sex**	The odds ratio of females to males: 1.046 (95% CI: 0.712–1.539, *P* = 0.818); Heterogeneity: I^2^: 99.19%, *P* < 0.001							

*CI* Confidence intervals, *N* number

### The prevalence of regurgitation by gender

The daily, weekly, monthly, and overall prevalence of regurgitation in Iranian males was 3.59% (95% CI: 1.17–10.47%; heterogeneity: I^2^ = 97.58%, *P* <  0.001; *N* = 4788), 7.93% (95% CI: 4.55–13.46%; heterogeneity: I^2^ = 95.25%, *P* <  0.001; *N* = 5008), 10.15% (95% CI: 5.61–17.70%; heterogeneity: I^2^ = 97.28%, *P* <  0.001; *N* = 4788) and 28.00% (95% CI: 24.66–31.60%; heterogeneity: I^2^ = 81.76%, *P* <  0.001; *N* = 4788) (Figure 10-supplementary).

The daily, weekly, monthly, and overall prevalence of regurgitation in Iranian females was 4.63% (95% CI: 0.78–23.11%; heterogeneity: I^2^ = 98.76%, *P* <  0.001; *N* = 2803), 6.81% (95% CI: 3.64–12.41%; heterogeneity: I^2^ = 94.86%, *P* <  0.001; *N* = 3183), 5.23% (95% CI: 1.11–21.34%; heterogeneity: I^2^ = 98.49%, *P* <  0.001; *N* = 2803) and 30.59% (95% CI: 17.89–47.14%; heterogeneity: I^2^ = 98.29%, *P* <  0.001; *N* = 2803) (Figure 11-supplementary).

OR for the prevalence of daily, weekly, monthly, and overall prevalence of regurgitation in women compared to men in Table 4 shows that there is no significant difference in the prevalence of regurgitation.

### Meta-regression and publication bias for prevalence of regurgitation

The meta-regression model based on years of study for regurgitation prevalence revealed that the meta-regression coefficient for daily, weekly, monthly, and overall prevalence of regurgitation was (0.091, 95% CI: − 0.206 to 0.390, *P*= 0.546), (0.081, 95% CI: − 0.029 to 0.192, *P*= 0.149), (0.162, 95% CI: 0.027 to 0.297, *P* = 0.018) and (0.002, 95% CI: − 0.001 to 0.002, *P *< 0.001), respectively (Fig. 7).

Regarding publication bias, the significance level of Egger and Begg’s tests was (Egger = 0.060 and Begg’s = 0.452), (Egger = 0.221 and Begg’s = 0.999), (Egger = 0.011 and Begg’s = 0.999) and (Egger = 0.074 and Begg’s = 0.763) for the daily, weekly, monthly, and overall prevalence of heartburn, respectively (Figure 12-supplementary).

## Discussion

The present study is the first systematic review and meta-analysis on the prevalence of GERD in Iran. In this study, the prevalence of daily, weekly, monthly, and overall prevalence of GERD in Iranian population was 5.64%, 12.50%, 18.62%, and 43.07%, respectively. In a systematic review in 2014, the weekly prevalence of GERD in North America was 18.1–27.8%, in South America was 23.0%, in Europe was 8.8–25.9%, in East Asia was 2.5–7.8%, in Middle East was 8.7–33.1% and in Australia was 11.6%, and was specifically reported for Iran to be 10.1–15.0% [49], which is consistent with the present study.

In the present study, the causes of heterogeneity in the studies can be attributed to the geographic region and the studied population, while previous studies also mentioned racial and geographical factors for the pathogenesis of GERD [49, 50].

In a systematic review in Iran, the causes of heterogeneity for the prevalence of GERD have been attributed to different criteria such as definition, difference in social factors, cultural background, and lifestyle in different cities or different populations [51]. On the other hand, due to the limitations of population-based studies, where precise diagnostic methods such as PH metric testing cannot be used, some of these differences can be due to the lack of a comprehensive standard for classifying symptoms and complications of GERD, which makes comparison between studies difficult [52]. Some differences in reported reflux rates may be due to cultural and ethnic differences in perceiving, expressing, and understanding symptoms of reflux. For example, there are differences in describing symptoms and diseases in some areas and among some ethnic groups, while other groups do not pay attention to the symptoms of the disease. It has been pointed out that different groups and cultures have different perceptions of the word “heartburn”. In a study in Boston among different ethnic groups, only 13% of Chinese and Korean people had a proper understanding of the word “heartburn” [53].

Iranian people are gaining weight such that the prevalence of obesity in Iranian adults is 21.5% [54]. Meanwhile, the economic and social status of people has changed rapidly. Therefore, some studies have reported that the above factors are important risk factors [55].

Smoking has always been associated with GERD. The relationship between smoking and GERD (any symptoms) continues even after smoking is stopped [39]. Smoking increases the frequency of GERD by reducing the pressure of the sphincter [56] and decreases the secretion of the bicarbonate of the saliva [57]. However, some other mechanisms may also be involved in the relationship between smoking and symptoms of GERD. Therefore, smoking may result in exaggerated negative intrathoracic pressure and inspiratory thoraco-abdominal pressure gradient, which may cause gastrointestinal reflux [58, 59]. In a meta-analysis, the prevalence of smoking among Iranian men and women was reported to be 21.7% and 3.6%, respectively [59].

There is varied evidence regarding the relationship between gender and GERD symptoms, but most studies show no relationship [60]. However, in many studies based on endoscopy, non-erosive and erosive GERD are more common in men and women, respectively [61, 62]. In the present study, only the daily symptoms of GERD were significantly higher in women compared to men.

The prevalence of GERD-related symptoms and tissue damage is different in ethnic/racial groups [63, 64]. We found a significant difference between the weekly and overall prevalence of GERD in different areas; the weekly and overall prevalence of GERD in the south was 21.26% and in the north was 60.86%. Iran has different ethnicities (Kurds, Persians, Turks, Arabs, Turkmen, etc.) with different customs and lifestyles, each of which predominantly lives in certain geographic area (e.g., Kurds are concentrated in western Iran) [65]. Nevertheless, the environmental or genetic factors that affect these differences are not clear yet [39].

The study with highest quality in this meta-analysis was the study of Islami et al. [39] on 49,975 people of the general population, with a daily, weekly, monthly, and overall GERD prevalence of 11.83%, 8.06%, 40.96%, and 60.86%, respectively, who reported a high incidence.

In the present study, the prevalence of daily, weekly, monthly, and overall prevalence of GERD did not change significantly over time. In 2005, a systematic review on population-based studies reported the weekly prevalence of GERD to be 10–20% in Europe and the United States and less than 5% in East Asia [66]. However, in a more recent systematic review in 2011, the weekly prevalence of GERD was reported to be 8.8–25.9% in Europe and 18.1–27.8% in North America and 2.5–7.8% in East Asia 49). Therefore, the global prevalence of GERD is increasing over time [49].

The results of the Egger’s test show that bias has been suggested for the overall prevalence of GERD. Publication bias is usually suggested for studies that are based on relationship assessment scale because studies with a positive result are more likely [48, 67].

There were several limitations for this early study, so interpreting the results should be done with cautious. The questionnaire consisted of only the major and common symptoms of GERD such as heartburn and acid reflux, but not other symptoms. Non-gastric manifestations of GERD are not included. Indeed, in the absence of a golden standard for the diagnosis of GERD, we only have the questionnaires, which are common in clinical or epidemiological studies.

## Conclusion

The present meta-analysis provides comprehensive and useful information on the epidemiology of GERD in Iran for policy-makers and health care providers. This study showed a high prevalence of GERD in Iran. Therefore, effective measures on GERD-related factors such as lifestyle can be among the health policies of Iran.

## Supplementary information

**Additional file 1: Figure 1- supplementary**: The sensitivity analysis for daily (A), weekly (B), monthly (C), and overall (D) prevalence of GERD symptoms in Iranian population.

**Additional file 2: Figure 2-supplementary**: The daily (A), weekly (B), monthly (C), and overall (D) prevalence of GERD symptoms in Iranian males.

**Additional file 3: Figure 3-supplementary**: The daily (A), weekly (B), monthly (C), and overall (D) prevalence of GERD symptoms in Iranian females.

**Additional file 4: Figure 4-supplementary**: Publication bias for daily (A), weekly (B), monthly (C), and overall (D) prevalence of GERD symptoms.

**Additional file 5: Figure 5- supplementary**: The sensitivity analysis for daily (A), weekly (B), monthly (C), and overall (D) prevalence of heartburn in Iranian population.

**Additional file 6: Figure 6-supplementary**: The daily (A), weekly (B), monthly (C), and overall (D) prevalence of heartburn in Iranian males.

**Additional file 7: Figure 7-supplementary**: The daily (A), weekly (B), monthly (C), and overall (D) prevalence of heartburn in Iranian females.

**Additional file 8: Figure 8-supplementary**: Publication bias for daily (A), weekly (B), monthly (C), and overall (D) prevalence of heartburn.

**Additional file 9: Figure 9- supplementary**: The sensitivity analysis for daily (A), weekly (B), monthly (C), and overall (D) prevalence of regurgitation in Iranian population.

**Additional file 10: Figure 10-supplementary**: The daily (A), weekly (B), monthly (C), and overall (D) prevalence of regurgitation in Iranian males.

**Additional file 11: Figure 11-supplementary**: The daily (A), weekly (B), monthly (C), and overall (D) prevalence of regurgitation in Iranian females.

**Additional file 12: Figure 12-supplementary**: Publication bias for daily (A), weekly (B), monthly (C), and overall (D) prevalence of regurgitation.

**Additional file 13.** PRISMA 2009 Checklist.

## Data Availability

The datasets supporting the conclusions of this research are contained in the article.

## Abbreviations

GERD: Gastroesophageal reflux disease
NSAIDs: Nonsteroidal Anti-inflammatory Drugs
BMI: Body mass index
IranDoc: Iranian Research Institute for Information Science and Technology
SID: Scientific Information Database
RICST: Regional Information Center for Science and TechnologyMOOSE Meta-analyses Of Observational Studies in Epidemiology
PRISMA: Systematic Reviews and Meta-analysis
NOS: Newcastle Ottawa Scale
OR: Odds ratio
CI: Confidence interval
CMA: Comprehensive Meta-Analysis
