# Supplementary figures and images for: Epidemiology of gastroesophageal reflux disease in Iran: a systematic review and meta-analysis
Source: BMC Gastroenterol. 2020 Sep 14;20:297. doi: 10.1186/s12876-020-01417-6 (PMC7488684; doi:10.1186/s12876-020-01417-6)

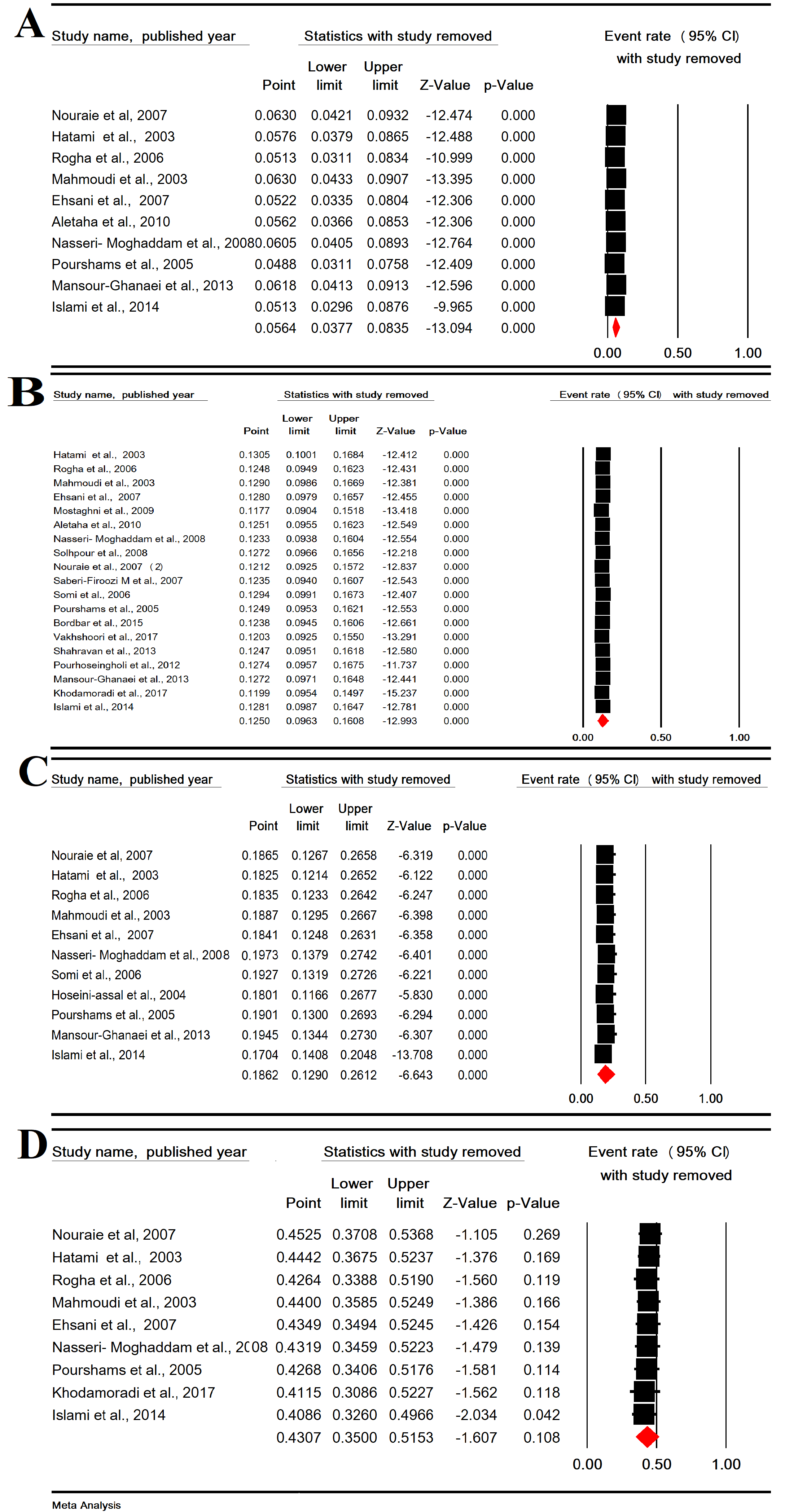

Supplement: Supplementary file 1 — Additional file 1: Figure 1- supplementary: The sensitivity analysis for daily (A), weekly (B), monthly (C), and overall (D) prevalence of GERD symptoms in Iranian population. [file 12876_2020_1417_MOESM1_ESM.tif]

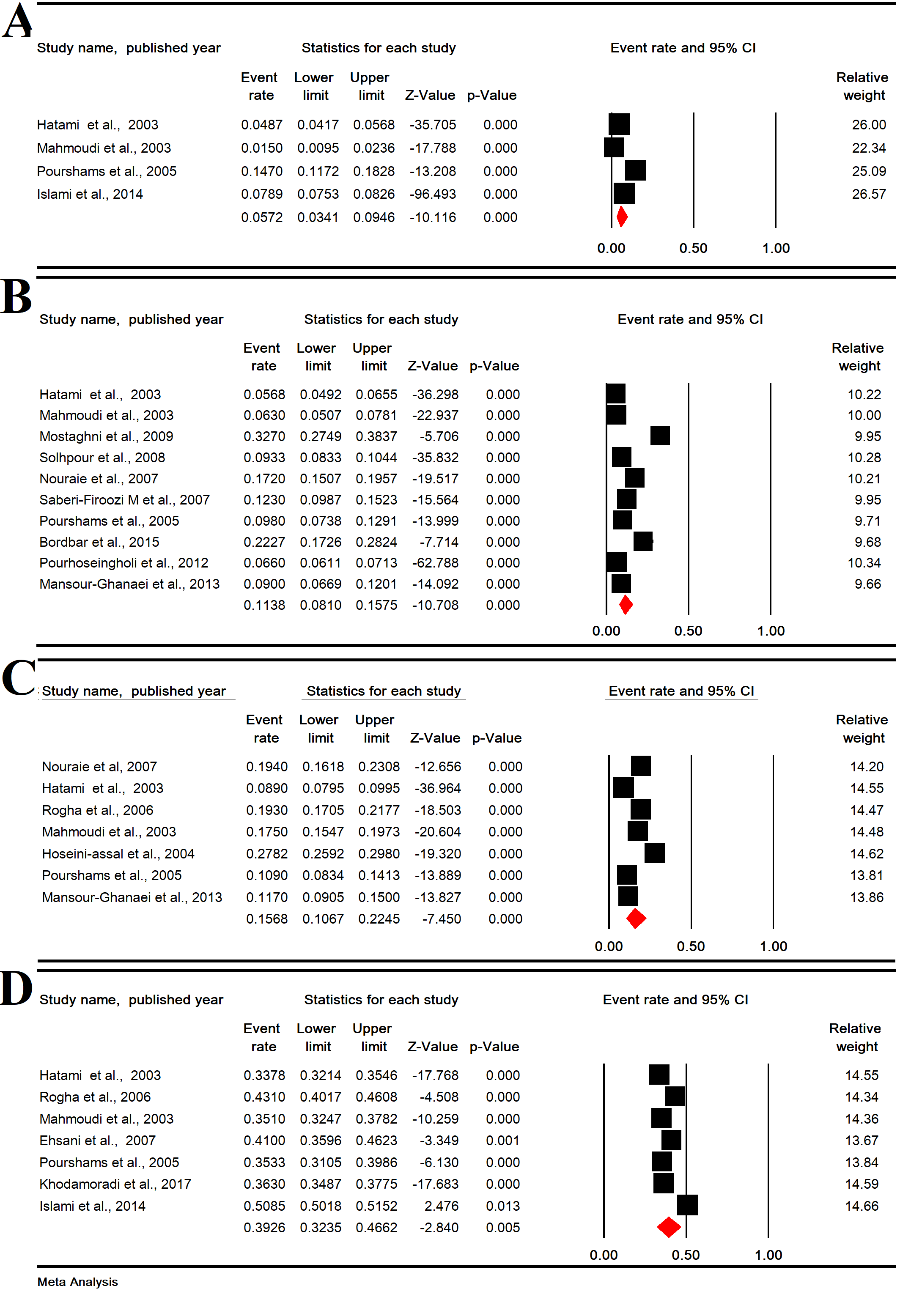

Supplement: Supplementary file 2 — Additional file 2: Figure 2-supplementary: The daily (A), weekly (B), monthly (C), and overall (D) prevalence of GERD symptoms in Iranian males. [file 12876_2020_1417_MOESM2_ESM.tif]

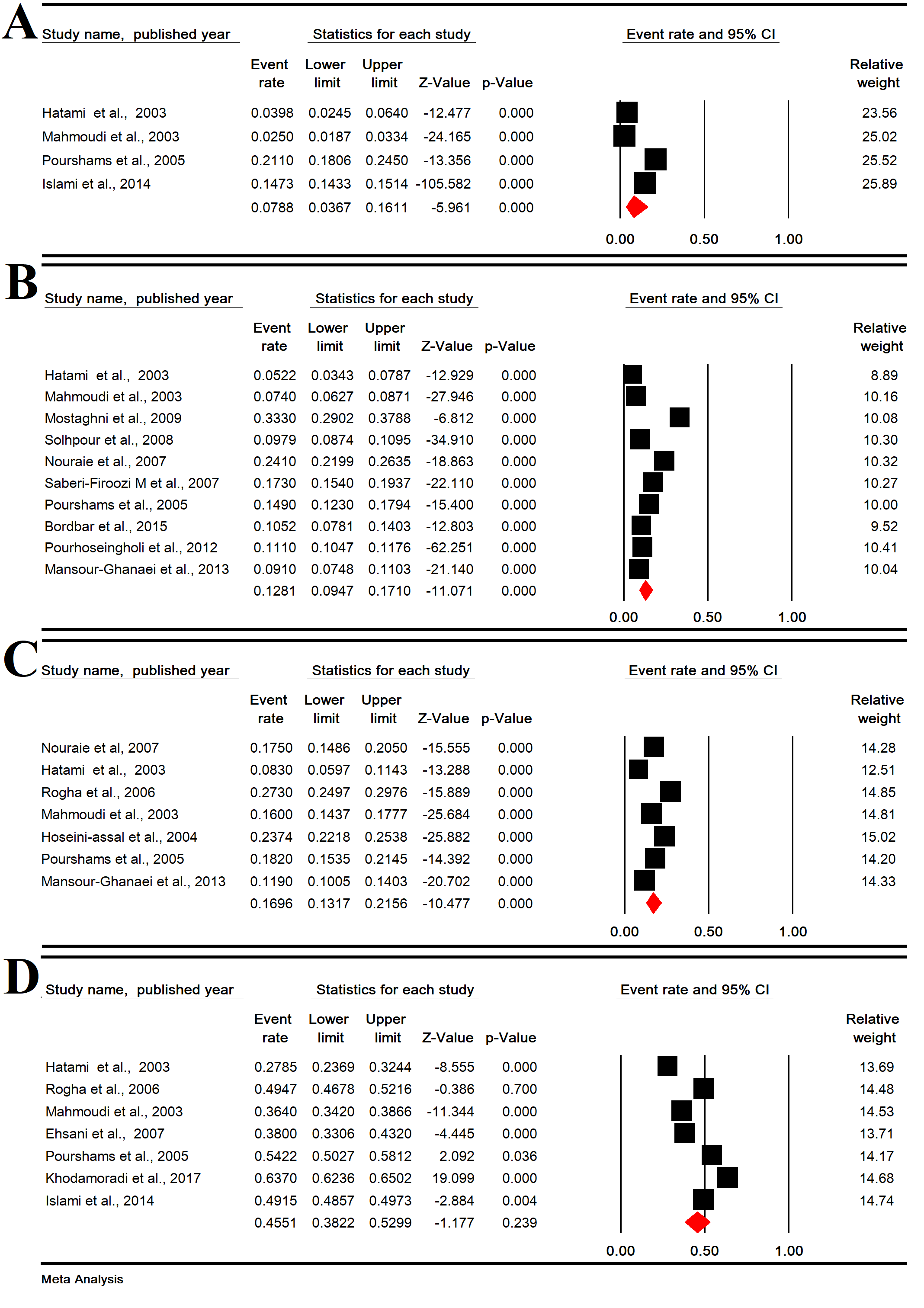

Supplement: Supplementary file 3 — Additional file 3: Figure 3-supplementary: The daily (A), weekly (B), monthly (C), and overall (D) prevalence of GERD symptoms in Iranian females. [file 12876_2020_1417_MOESM3_ESM.tif]

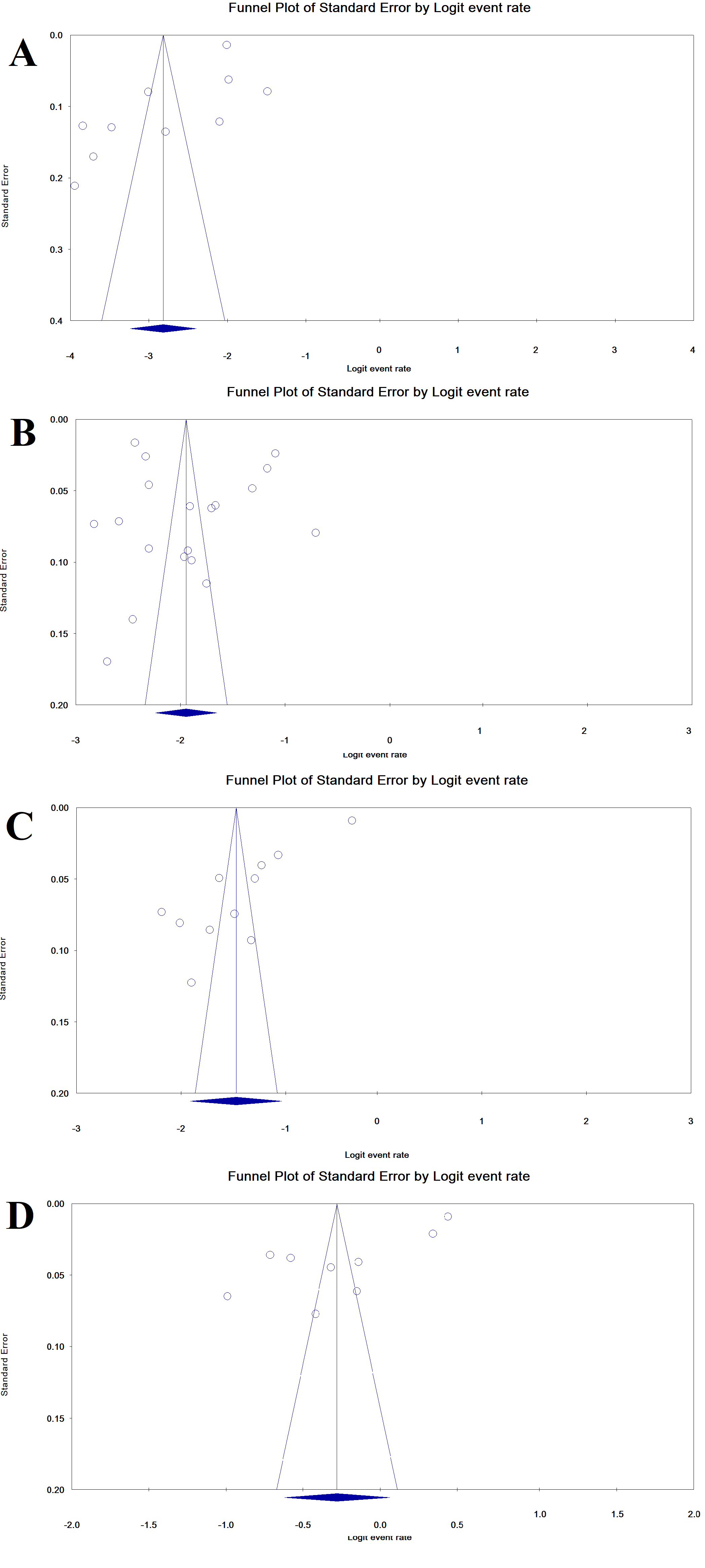

Supplement: Supplementary file 4 — Additional file 4: Figure 4-supplementary: Publication bias for daily (A), weekly (B), monthly (C), and overall (D) prevalence of GERD symptoms. [file 12876_2020_1417_MOESM4_ESM.tif]

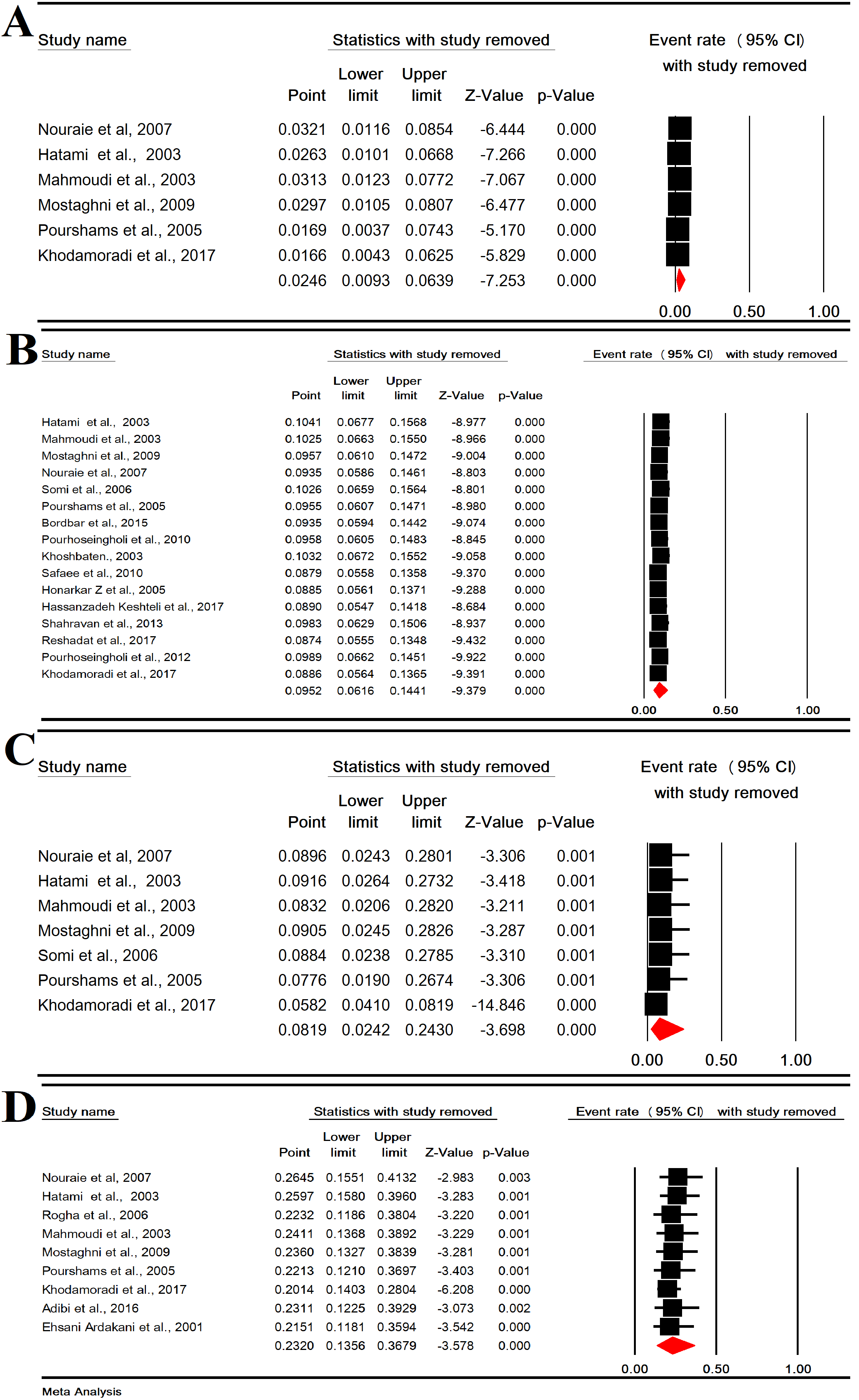

Supplement: Supplementary file 5 — Additional file 5: Figure 5- supplementary: The sensitivity analysis for daily (A), weekly (B), monthly (C), and overall (D) prevalence of heartburn in Iranian population. [file 12876_2020_1417_MOESM5_ESM.tif]

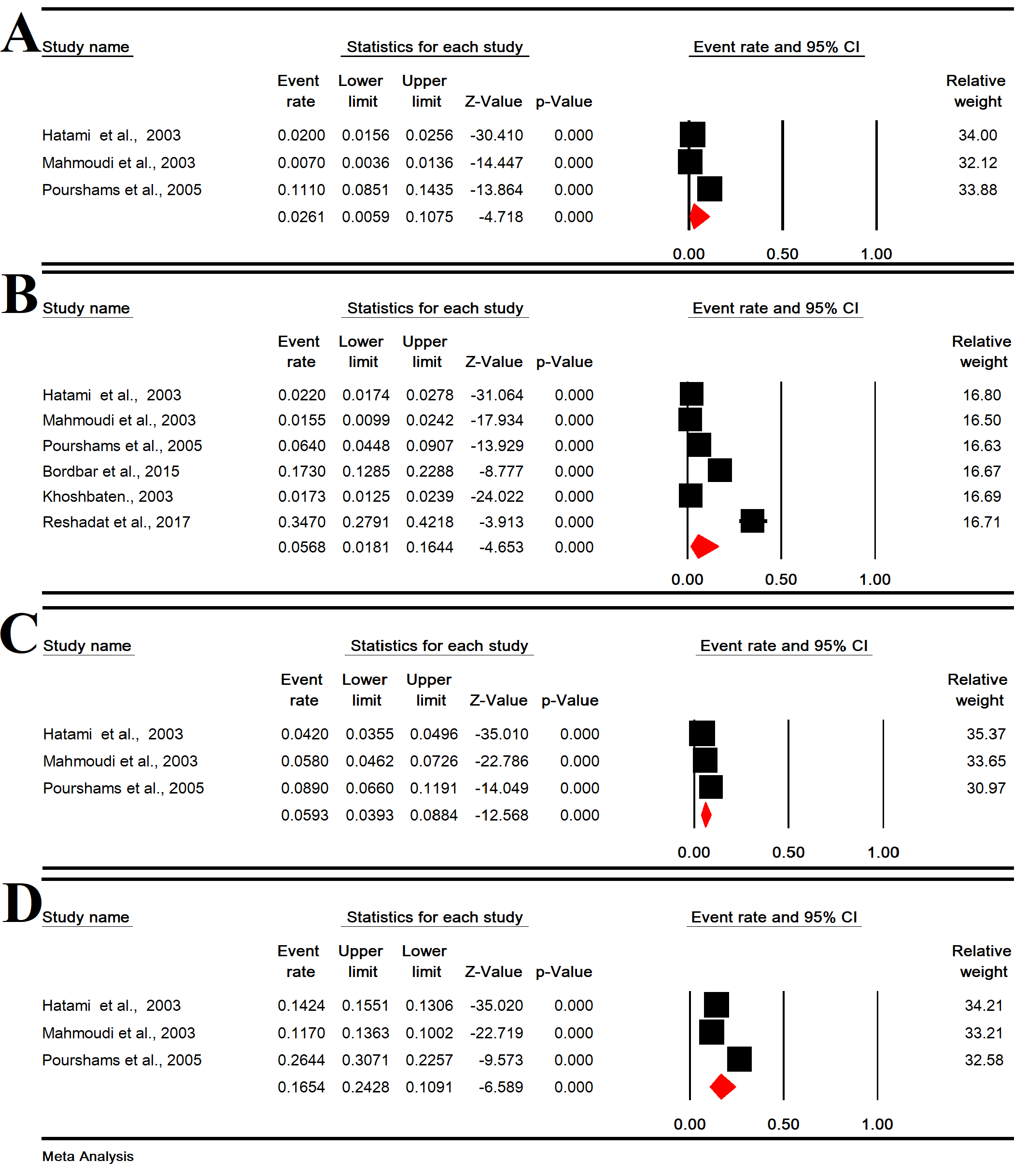

Supplement: Supplementary file 6 — Additional file 6: Figure 6-supplementary: The daily (A), weekly (B), monthly (C), and overall (D) prevalence of heartburn in Iranian males. [file 12876_2020_1417_MOESM6_ESM.tif]

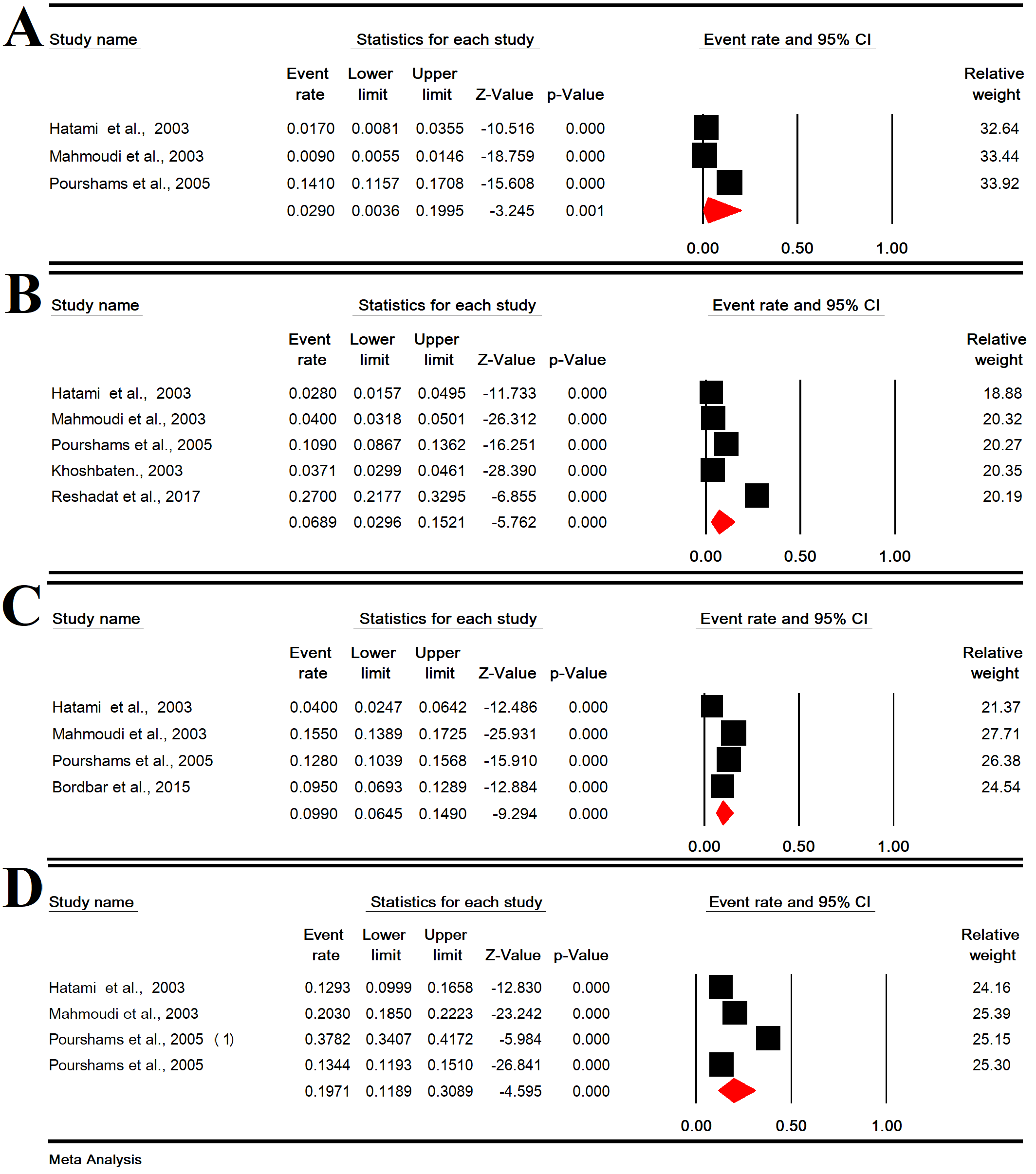

Supplement: Supplementary file 7 — Additional file 7: Figure 7-supplementary: The daily (A), weekly (B), monthly (C), and overall (D) prevalence of heartburn in Iranian females. [file 12876_2020_1417_MOESM7_ESM.tif]

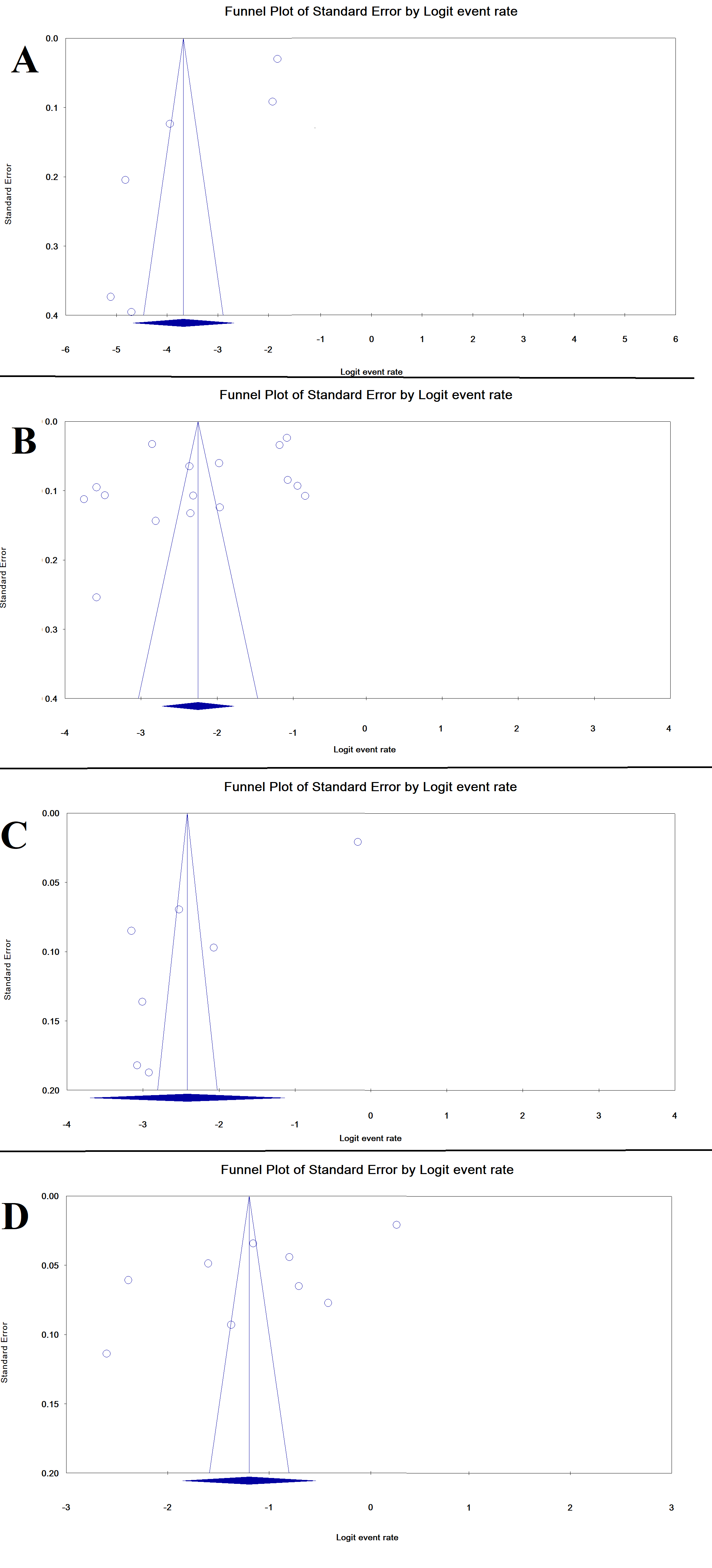

Supplement: Supplementary file 8 — Additional file 8: Figure 8-supplementary: Publication bias for daily (A), weekly (B), monthly (C), and overall (D) prevalence of heartburn. [file 12876_2020_1417_MOESM8_ESM.tif]

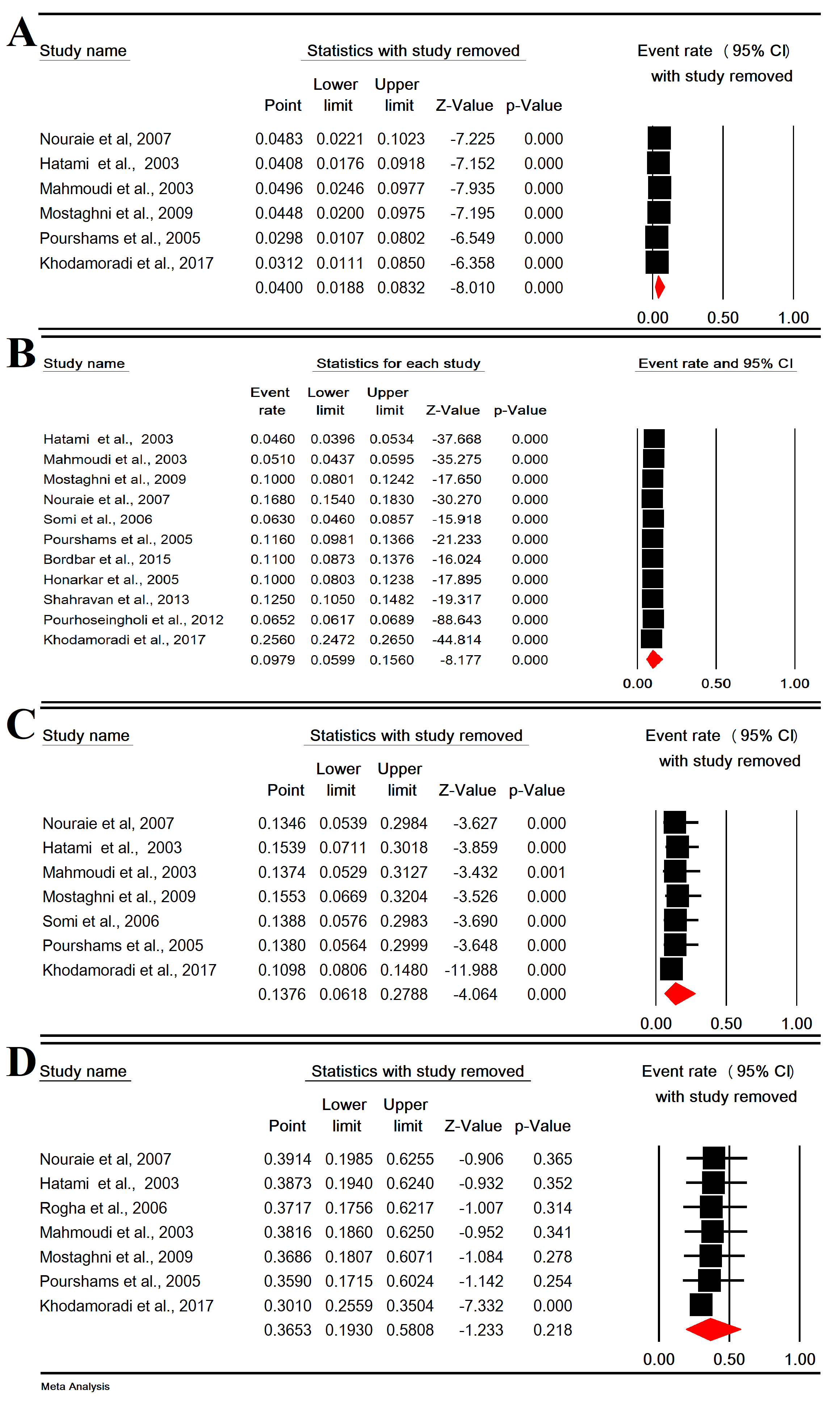

Supplement: Supplementary file 9 — Additional file 9: Figure 9- supplementary: The sensitivity analysis for daily (A), weekly (B), monthly (C), and overall (D) prevalence of regurgitation in Iranian population. [file 12876_2020_1417_MOESM9_ESM.tif]

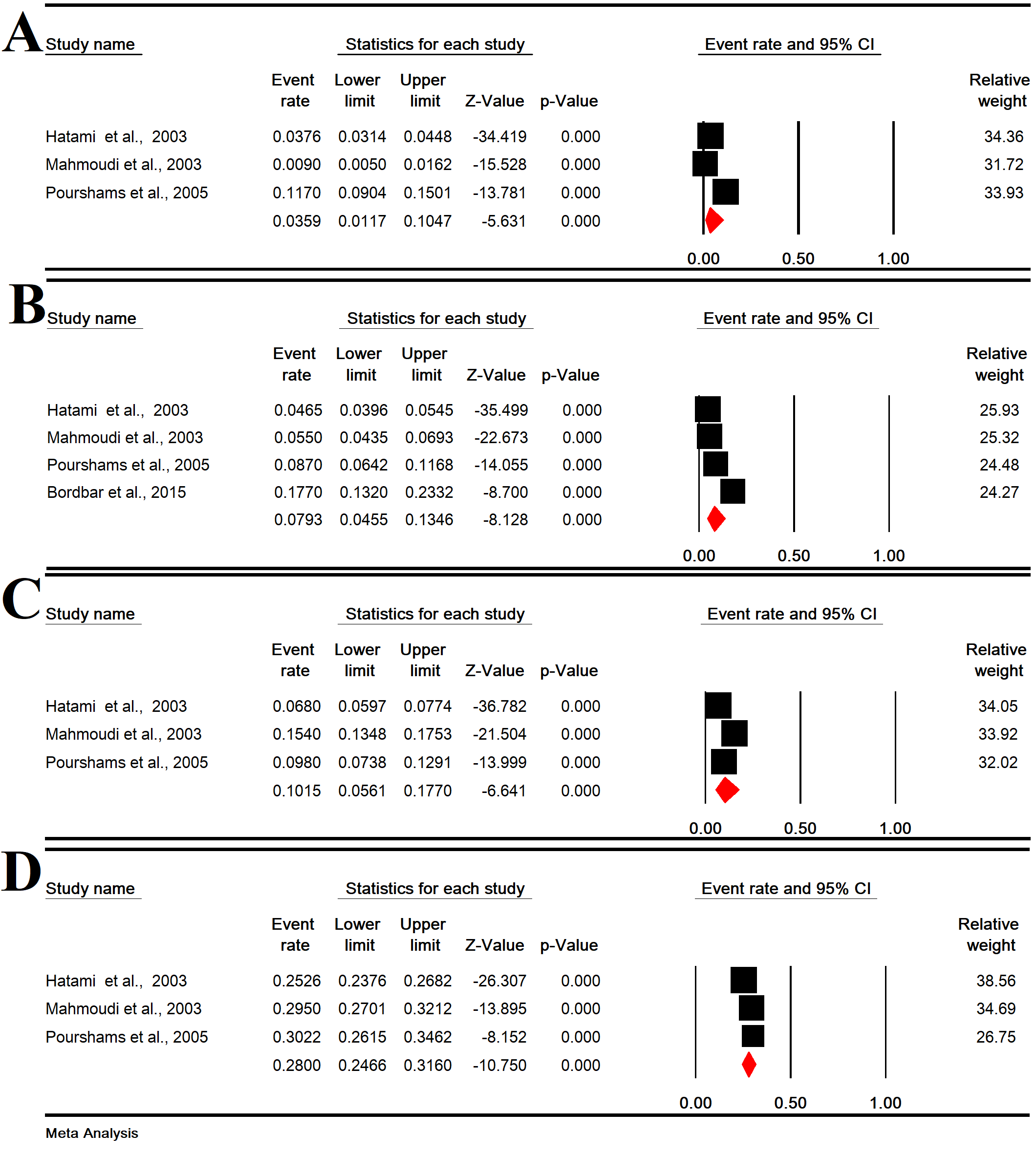

Supplement: Supplementary file 10 — Additional file 10: Figure 10-supplementary: The daily (A), weekly (B), monthly (C), and overall (D) prevalence of regurgitation in Iranian males. [file 12876_2020_1417_MOESM10_ESM.tif]

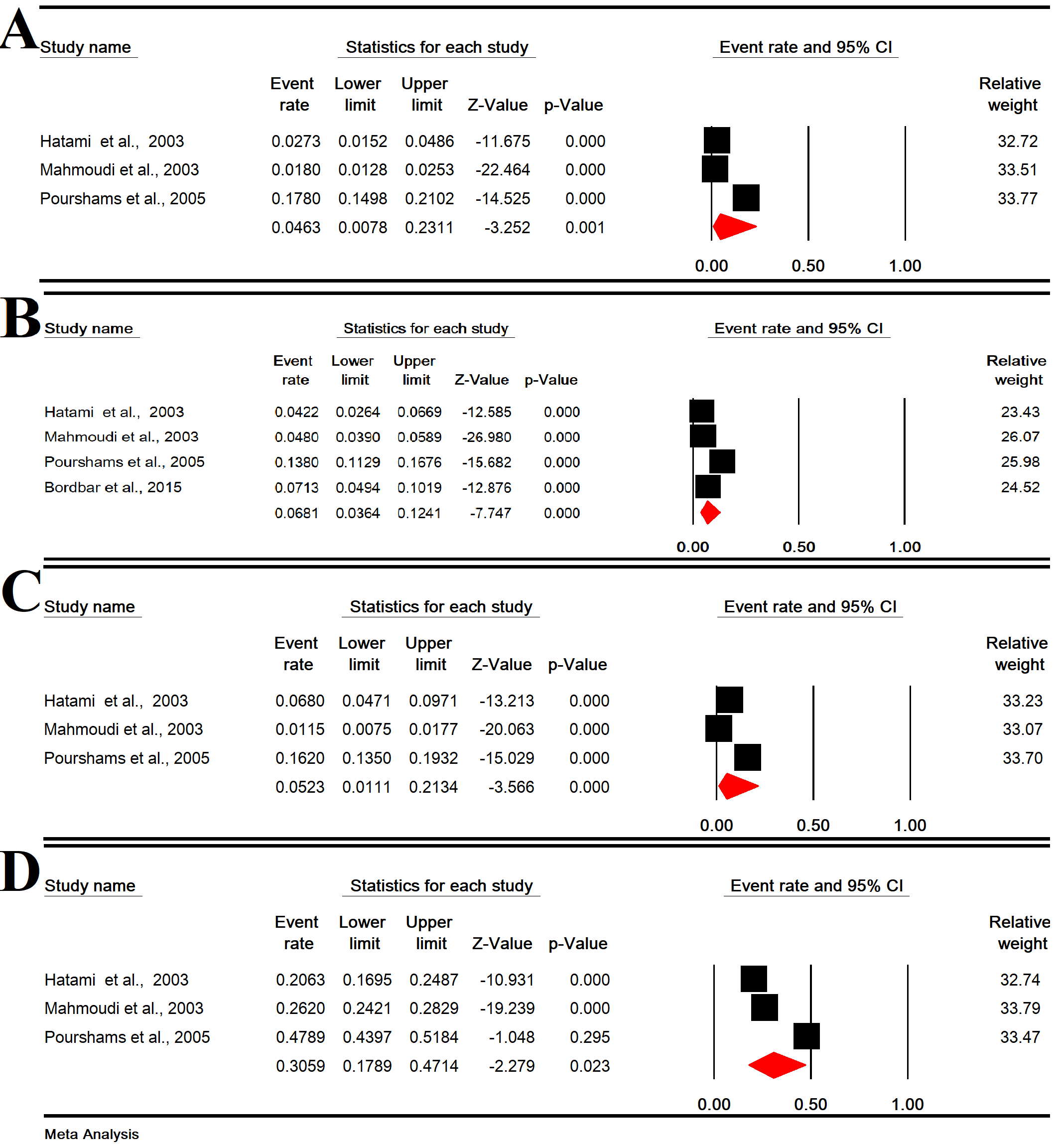

Supplement: Supplementary file 11 — Additional file 11: Figure 11-supplementary: The daily (A), weekly (B), monthly (C), and overall (D) prevalence of regurgitation in Iranian females. [file 12876_2020_1417_MOESM11_ESM.tif]

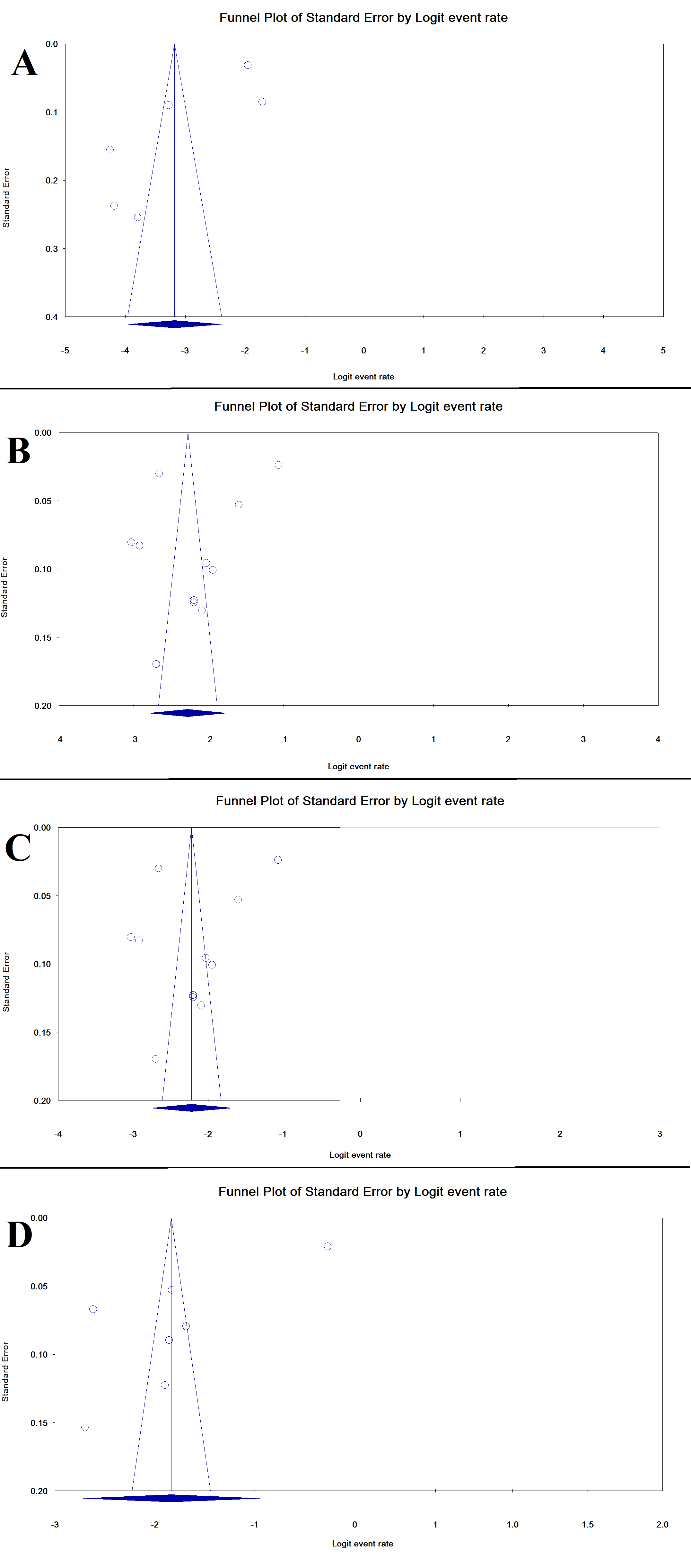

Supplement: Supplementary file 12 — Additional file 12: Figure 12-supplementary: Publication bias for daily (A), weekly (B), monthly (C), and overall (D) prevalence of regurgitation. [file 12876_2020_1417_MOESM12_ESM.tif]
